# Duality in disease: How two amino acid substitutions at actin residue 312 result in opposing forms of cardiomyopathy

**DOI:** 10.1016/j.jbc.2024.107961

**Published:** 2024-11-05

**Authors:** Karl E. Steffensen, Michael R. Jones, Elma Misini, Chloe J. King, Andrea Pace, John F. Dawson

**Affiliations:** Department of Molecular and Cellular Biology, University of Guelph, Guelph, Ontario, Canada

**Keywords:** actin, ACTC, cardiomyopathy, hypertrophic cardiomyopathy, dilated cardiomyopathy, molecular dynamics, troponin, tropomyosin, myosin, myosin binding protein C, C0C2

## Abstract

Two common types of cardiovascular disease are hypertrophic cardiomyopathy (HCM) and dilated cardiomyopathy (DCM) which occur from changes to sarcomere contractile mechanisms and activity. Actin amino acid substitutions R312C and R312H have been found in HCM and DCM patients, respectively. Previously, we observed that R312C/H variants display both hyperactivity and hypoactivity *in vitro*, contradicting traditional characterizations of HCM- and DCM-causing variants. Here, we further characterized R312C/H actin variants *in vitro* and conducted *in silico* modeling to better understand the mechanisms differentiating HCM and DCM. Our results suggest that R312C/H substitutions cause structural changes that differentially impact actomyosin activity. A gradient of altered interactions with regulatory proteins troponin, tropomyosin, and the C0C2 domains of myosin-binding protein C was also observed, influencing the accessibility of active and inhibitory conformations of these proteins. The results presented here support our previous suggestion of a gradient of factors that differentiate between HCM and DCM. Further characterization of HCM- and DCM-causing actin variants using *in vitro* and *in silico* methods is required for better understanding cardiomyopathy and improving clinical outcomes.

Cardiovascular disease (CVD) has vast economic and social impacts (1). Heritable CVDs include cardiomyopathies (CM), a designation that encompasses hypertrophic cardiomyopathy (HCM) and dilated cardiomyopathy (DCM). HCM and DCM, classified as a thickening and dilation of the left ventricular myocardium respectively, can lead to systolic or diastolic dysfunction and potentially heart failure (2). The mechanisms leading to CM are not fully understood due to the wide range of behaviors for each CM-linked mutation, though HCM is associated with sarcomere hyperactivity and DCM with sarcomere hypoactivity. The significance of research into HCM and DCM is clear and understanding the mechanisms resulting in HCM and DCM is critical for improving clinical outcomes and relieving the economic burden of CVD.

Among the sarcomere proteins which have been linked to CM, cardiac actin (ACTC) is unique due to its high sequence conservation, with actin amino acid substitutions leading to both HCM and DCM (3, 4). Actin has numerous binding partners in the sarcomere, known as actin binding proteins (ABPs), including tropomyosin (Tm), troponin (Tn), myosin, and cardiac myosin-binding protein C (cMyBP-C). Actin’s interactions act as the foundation for sarcomere contraction. Under low calcium conditions, Tm blocks myosin-binding sites on the actin filament in what is known as the blocked (B) state (5). Calcium binding to Tn induces structural changes that pull on Tm, exposing myosin-binding sites in what is known as the closed (C) state (5). Upon myosin binding to actin, Tm is pushed across actin’s surface into the myosin-bound open (M) state (5), where the actomyosin complex generates contractile forces. The C0C2 domains of cMyBP-C bind to actin and shift Tm to help thin filament activation (6, 7, 8, 9), increasing force production as well as constitutive activity under low calcium while decreasing activity under high calcium *in vitro* (9, 10). Actin amino acid substitutions disrupt actin:ABP interactions, altering the intricacies of sarcomere contraction and resulting in diseases such as CM.

Amino acid substitutions at ACTC residue 312 have been found in patients with different forms of cardiomyopathy. Though the pathogenicity is difficult to determine, R312C was found in patients clinically presenting with HCM (4), while R312H has been found in both HCM and DCM patients (3) which possibly positions it in the subset of variants that progress from HCM to DCM (11). Residue 312 is located at the interface of actin subdomains 3 and 4 (SD3/4) (Fig. 1*A*), forming stabilizing interactions (Fig. 1*B*) within a region of F-actin (Fig. 1, *C* and *D*) that plays a central role in Tm binding (Fig. 1*E*). One theory in the field is that HCM hyperactivity and DCM hypoactivity result from increased and decreased calcium sensitivity, respectively, altering Tm/Tn regulation (12, 13); however, the causes of cardiomyopathies are complex and involve more than just calcium sensitivity. For example, the myosin R304Q mutation results in HCM but causes a decrease in myosin motor activity (14), suggesting hypoactivity; at the same time, the mutation leads to more myosin heads bound to the thin filament (15), suggesting hyperactivity.

**Figure 1:**
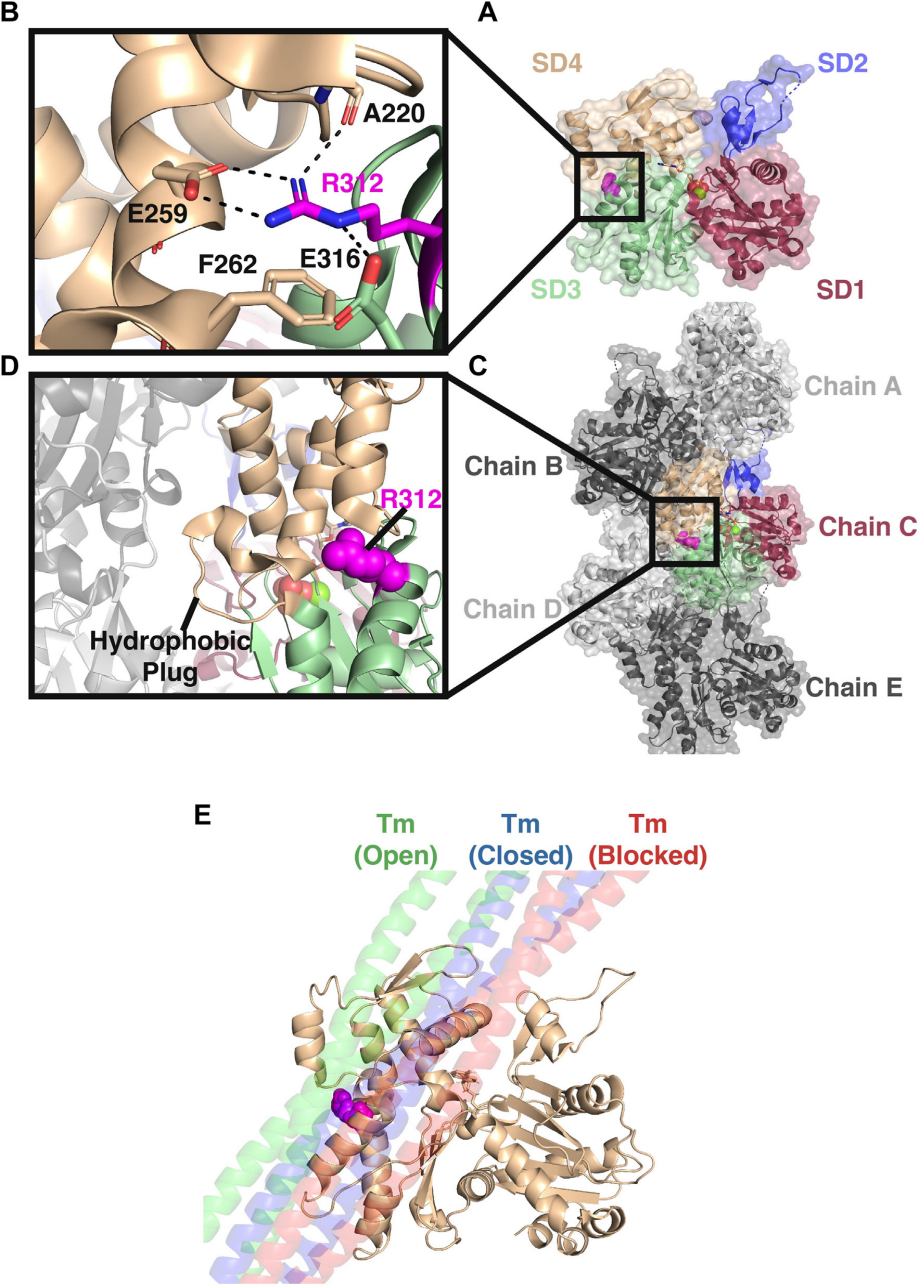
**Actin’s structure and substitutions at residue R312.***A*, the structure of monomeric G-actin, with its four subdomains (SD) colored (PDB 2BTF). *B*, the location of residue 312 (colored *magenta*) and the stabilizing interactions it forms. *C*, the structure of a 5 protomer polymeric actin filament, with a single protomer highlighted. *D*, the location of residue 312 (colored *magenta*) in relation to the neighboring F-actin protomers. *E*, the tropomyosin (Tm) binding conformations overlayed on an F-actin protomer with residue 312 (colored *magenta*) highlighted. Tm binds to actin filaments in the blocked (colored *red*, PDB 7UTL) state in the absence of calcium. In the presence of calcium, troponin pulls Tm into the closed (colored *blue*, PDB 7UTI) state. Upon myosin binding to F-actin, Tm is pushed into the open (colored *green*, PDB 8EFI) state.

We previously hypothesized that R312C/H would lead to actin structural changes throughout the Tm-binding region, resulting in hyperactivity for R312C and hypoactivity in R312H (16). We observed, however, that both variants displayed decreased calcium sensitivity *in vitro*, suggestive of hypoactivity, as well as increased activity at low calcium concentrations, suggestive of hyperactivity (16). Further, actomyosin force output was increased with R312C and decreased with R312H. Seemingly conflicting characteristics raised the question: how do two amino acid substitutions at the same residue result in differing CM progression, despite similar biochemical profiles *in vitro*? Due to the observed activity under relaxing, low calcium conditions and the location of actin residue 312 near the Tm-binding regions, there is evidence of altered interactions between actin and regulatory proteins Tn/Tm. Further, hyperactivity under unregulated conditions and altered actomyosin force output indicates changes to the actomyosin interaction. We therefore sought to study the basic contractile mechanism using actin variants R312C and R312H, clarifying how changes might differentiate between HCM *versus* DCM.

A variety of techniques were used to examine the effects of the R312C and R312H actin variants on ABP binding and regulatory functions *in vitro*. We observed a reduced rate of kinetic Tm binding as well as reduced Tm regulation of actomyosin interactions, in addition to hyperactivity under low-calcium conditions in the presence of C0C2. Structural models of actin R312C and R312H variants were developed *in silico*, identifying structural rearrangements to the ‘tropomyosin bumper’ (Tm-bumper, residues 222–230) and changes to internal communication networks unique to each variant. *In silico* results support our *in vitro* findings of similarly altered interactions with ABPs in R312C/H systems. Changes in Tm-bumper movement explains decreased Ca^2+^ sensitivity at higher pCa, while opposing shifts of actin’s DNaseI-binding loop (D-loop, residues 40–50) in R312C and R312H differentially impacts actomyosin interactions and force output. Further characterization of HCM- and DCM-causing actin variants to identify the differentiating mechanisms behind CM is critical for developing targeted treatments to improve clinical outcomes.

## Results

To test our hypothesis that variable interactions with actomyosin and regulatory proteins differentiate between HCM and DCM, we measured the impacts of R312C/H substitutions on Tm binding and regulatory functions, as well as regulation in the presence of C0C2 *in vitro*. *In silico* characterization provided details of the structural changes behind our *in vitro* observations.

### R312C/H substitutions alter tropomyosin activity

**Figure 2:**
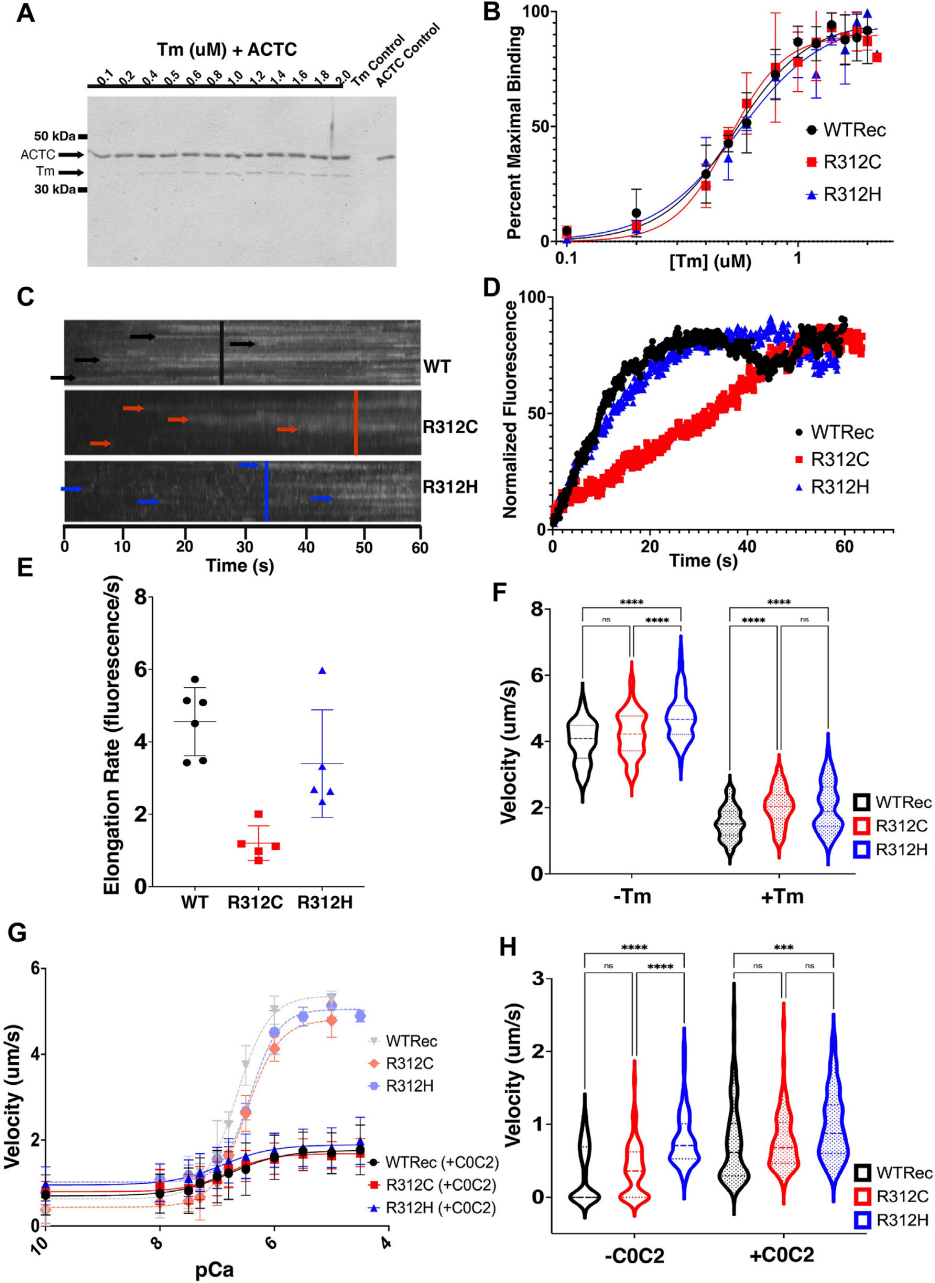
***In vitro* results.***A*, an SDS-PAGE for the R312C variant following a co-sedimentation assay. Actin bands are on *top*, and tropomyosin bands are on the *bottom*. Tropomyosin bands increase in intensity along with tropomyosin concentration. *B*, co-sedimentation assay curves, fit using a specific binding with Hill slope in Prism GraphPad, of 7 μM ACTC proteins in the presence of Tm ranging from 0.1 to 2 μM Tm. N = 3 where N is a single co-sedimentation curve. Error bars represent SD. Three independent protein purifications were studied, with each being utilized for a single replicate. The K_d_ are listed in Table 1. *C*, kymographs of Alexa488-labeled tropomyosin binding along the length of an actin filament. A line was drawn along a filament and intensity measured over 60 s, increasing as Tm binds along the filament. *Top*: kymograph with WTRec; *middle*: R312C; *bottom*: R312H. For each kymograph, arrows represent the approximate start of intensity measurements following tropomyosin binding and elongation. Vertical lines represent the approximate start of peak intensity measurements. *D*, an average of all fluorescence increases over time, normalized from 1 to 100 percent. The time when fluorescence begins to increase (tropomyosin begins to bind) is counted as timepoint “0.” Three different purifications were utilized for rate measurements, with two purifications utilized for R312C, with one video per purification. 1 to 3 filaments were measured per video, with each filament being counted as a single N value. WTRec had N = 6, R312C had N = 5, and R312H had N = 5. *E*, elongation rates (slope) of the binding curves were assessed from 0 to 15 s and plotted as a scatterplot displaying the mean, and error bars representing SD. Statistical significance was determined with a one-way Anova, followed by a *post hoc* Tukey test. The mean elongation rate for R312C was significantly different than WTRec (*p* = 0.0004) and R312H (*p* = 0.0142). The mean elongation rate for R312H was not significantly different than WTRec (*p* = 0.1961). *F*, violin plots displaying motility of ACTC filaments in the absence (−Tm) and presence (+Tm) of tropomyosin (N = 90, where N is an individual filament), with lines representing the median and quartiles. Statistical analyses were performed with one-Way Anova w/Post-hoc Tukey Tests as indicated by the brackets above the violin plots, where statistical significance is indicated by ∗ and no significance by ns. In the absence of tropomyosin, the average velocity of R312H filaments was significantly higher than WTRec and R312C. In the presence of tropomyosin, the average velocity of R312H and R312C filaments were significantly higher than WTRec. *G*, *in vitro* motility assays in the absence and presence of C0C2 where N = 9 and N is equivalent to the average of 10 filament gliding velocities from one video at each pCa concentration. Three separate actin purifications were studied, with each purification being measured with three distinct videos. Error bars represent SD. Statistical analyses were conducted using Kruskal–Wallis w/Dunn’s Multiple Comparisons Test. Following the addition of C0C2, V_max_ was decreased for WTRec and both R312 variants as expected, with an observed trend of hyperactivity for the R312H variant across all calcium concentrations. For R312C, there was no significant difference compared to WTRec for pCa_50_ (*p* = 0.9897) and V_max_ (*p* > 0.999). For R312H, there was no significant difference compared to WTRec for pCa_50_ (*p* = 0.9129) and V_max_ (*p* > 0.999). *H*, violin plots of gliding velocities for actin filaments at pCa 10 in the absence and presence of C0C2 (N = 90, where N is an individual filament), with lines representing the median and quartiles. Three distinct actin purifications were studied with 30 filaments of each purification being included in the measurements. Statistical analyses were conducted using Kruskal–Wallis w/Dunn’s Multiple Comparisons Test, represented by brackets above the violins. Statistical significance is noted by ∗, while no significance is noted by ns. In the absence of C0C2, the average velocity for R312H is significantly higher than WTRec (*p* < 0.0001) and R312C (*p* < 0.0001) but was not significant between R312C and WTRec (*p* > 0.9999). In the presence of C0C2, the average gliding velocity for R312H was significantly higher than WTRec (*p* = 0.0005), but not R312C (*p* = 0.1515).

Alterations to the actin:Tm interaction in the absence of other regulatory proteins was measured through equilibrium co-sedimentation and kinetic binding assays (Fig. 2, *A* and *B*). In the co-sedimentation assay, no major differences in K_d_ values were observed between the R312 variants and WTRec (Table 1), contrary to our hypothesis that the R312 variants would disrupt Tm binding to actin filaments (16).

**Table 1:** Compiled assay results

Experimental value	WTRec	R312C	R312H
Tm Co-sedimentation			
K_d_ (uM)	0.533 ± 0.114	0.505 ± 0.141	0.555 ± 0.128
Tm Elongation			
Elongation Rate (/s)	4.561 ± 0.942	1.199 ± 0.482	3.397 ± 1.488
Tm Inhibition			
Velocity (−Tm, um/s)	3.99 ± 0.626	4.229 ± 0.676	4.733 ± 0.673
Velocity (+Tm, um/s)	1.545 ± 0.479	2.036 ± 0.554	2.023 ± 0.723
C0C2 IVM			
V_max_ (um/s)	1.77 ± 0.580	1.69 ± 0.328	1.89 ± 0.551
V_min_ (um/s)	0.724 ± 0.521	0.768 ± 0.382	0.980 ± 0.466
Hill Coefficient	0.931 ± 0.49	1.16 ± 0.48	0.991 ± 0.49
pCa_50_	6.77 ± 0.257	6.82 ± 0.162	6.90 ± 0.237

Tabulation of results for the ACTC variants R312C and R312H from *in vitro* assays. Values are listed as the average ± SD. Three ACTC purifications were utilized three times for data collection. To determine which statistical test to use, normality of each data set was determined with Shapiro–Wilk Tests. For the Tm co-sedimentation assay, N = 3 where N is equivalent to a normalized densitometry result from the Tm bands in each respective SDS-PAGE gel. When measuring Tm elongation rates, WTRec-ACTC had N = 6, R312C-ACTC and R312H-ACTC had N = 5. The elongation rate was significantly different between WTRec *versus* R312C and R312C *versus* R312H but was not significantly different between WTRec *versus* R312H (one way ANOVA w/*post hoc* Tukey Test). Velocities were measured in the presence and absence of Tm, with N = 90 where N is the measurement of an individual filament velocity. In the absence of Tm, the average velocity was significantly different between WTRec *versus* R312H and R312C *versus* R312H, but not significantly different between WTRec *versus* R312C (one way ANOVA w/*post hoc* Tukey Test). In the presence of Tm, the average velocity was significantly different between WTRec *versus* R312C and WTRec *versus* R312H but was not significantly different between R312C *versus* R312H (one way ANOVA w/*post hoc* Tukey Test). For the IVM assay containing C0C2, N = 9 where N is equivalent to the average of 10 filaments in a video. The V_max_ and Hill Coefficient in the IVM assay of the R312 variants was not statistically different to that seen for WTRec (Kruskal–Wallis w/Dunn’s Multiple Comparisons Test). The R312 variants were not significantly different from WTRec for their pCa_50_ values (Kruskal–Wallis w/Dunn’s Multiple Comparisons Test) but trended towards a leftward shift of the pCa_50_ values. The average velocity at pCa_10_ (V_min_) was significantly different for R312H *versus* WTRec, but there was no significant difference between R312C *versus* WTRec or R312C *versus* R312H (Kruskal–Wallis w/Dunn’s Multiple Comparisons Test).

The rate of Tm binding under kinetic conditions was also measured with TIRF microscopy (Fig. 2, *C* and *D*). The rate of tropomyosin binding was lower for the R312H and R312C filaments than that for WTRec (Fig. 2*E*, Table 1). The elongation rate for R312C was significantly different from that of WTRec (*p* < 0.05, one way ANOVA w/*post hoc* Tukey Test). R312H did not display a statistically different binding rate than WTRec but continued the trend towards a lower elongation rate. Taken together, the equilibrium binding of Tm to R312C/H filaments is unaltered, but the rate of binding is reduced.

In the absence of Tn, Tm sterically blocks the myosin-binding sites on actin, inhibiting actin filament velocities *in vitro* (17, 18). Due to the position of the R312C/H substitutions, as well as the observed alterations to the kinetic binding rate, we tested F-actin motility in the presence of Tm without Tn to measure changes to Tm’s inhibitory activity (17, 18). Unregulated filament motility under maximal activity was first measured in the absence of Tm (Table 1). Confirming our previous results (16), the average velocity of R312H filaments was significantly higher than WTRec (*p* < 0.05, one way ANOVA w/*post hoc* Tukey Test) (Fig. 2*F*) while the R312C velocity was not significantly different than WTRec. Upon the addition of Tm, filament velocity was reduced as expected (Table 1). Notably, the velocities of both R312H and R312C filaments in the presence of Tm were significantly higher than that of WTRec (*p* < 0.05, one way ANOVA w/*post hoc* Tukey Test) (Fig. 2*F*), indicating a reduced ability of Tm to inhibit myosin activity in R312 variant filaments.

To this point, we observed that both variants display reductions in calcium sensitivity (16), rates of kinetic Tm binding, and inhibitory activity of Tm, alongside increased activity under low calcium concentrations, all suggesting that R312C/H may experience similarly altered interactions with regulatory proteins Tm/Tn. The C0C2 domains of cMyBP-C play a role in Tm positioning and regulation, affecting calcium sensitivity and filament velocity *in vitro*. Given our observed alterations to Tm activity, we conducted in vitro motility (IVM) assays with the addition of C0C2 to determine if its regulation was similarly affected, potentially clarifying functional differences that might elucidate the mechanisms behind HCM *versus* DCM.

### C0C2 has variable impact on R312C/H thin filament activity

To further detect changes in thin filament regulation, a variation of the IVM assay was conducted. We measured the change in the velocity of regulated thin filaments with Tn/Tm bound in the presence of the C0C2 regulatory protein (Table 1). Previously, regulated thin filament velocities decreased while calcium sensitivity increased in the presence of C0C2 (19), which was observed here as the V_max_ of all three filament types was lower than that of previous assays in the absence of C0C2 (Fig. 2*G*). In the presence of C0C2, following the trend of our previous results (16), R312H displayed the greatest V_max_, while R312C displayed the lowest V_max_ (Table 1), though differences were not statistically significant (*p* > 0.05, Kruskal–Wallis w/Dunn’s Multiple Comparisons Test). R312H and R312C proteins displayed a trend of lower pCa_50_ than that of WTRec (Table 1), suggestive of hyperactive filaments.

As increased motility was previously observed for both variants in the absence of C0C2 under low calcium conditions at pCa 10^16^, motility at pCa 10 was again measured here in the presence of C0C2. R312H had the highest motility at pCa 10, followed by R312C, then WTRec (Fig. 2*H*). Differences in average pCa 10 velocities were significant for WT *versus* R312H (*p* < 0.05, Kruskal–Wallis w/Dunn’s Multiple Comparisons Test) but were not significant between WT *versus* R312C and R312C *versus* R312H (*p* > 0.05, Kruskal–Wallis w/Dunn’s Multiple Comparisons Test).

We found that the properties of C0C2 to broadly reduce filament velocity and increase calcium sensitivity (19) were unchanged, and the pattern of hypersensitivity under pCa 10 follows our previous results (16), further evidence of altered thin filament regulation under low calcium conditions. Hypersensitivity at all calcium concentrations for R312H, however, was unexpected. Thus far, our variants have demonstrated contradictory properties, displaying both hyperactivity and hypoactivity under different conditions, clouding the mechanisms contributing to disease. To understand the factors driving our *in vitro* results and identify mechanisms behind HCM *versus* DCM, we conducted *in silico* characterization to observe the structural changes resulting from R312C/H substitutions.

### Actin’s properties *in silico* change in R312C/H variants

Molecular dynamics (MD) simulations were carried out for WT, R312C, and R312H systems for monomeric G-actin (G-WT, G-R312C, and G-R312H) and polymeric F-actin (F-WT, F-R312C, and F-R312H) consisting of five subunits. Analyses were focused on F-actin systems, since no differences in the intrinsic properties of G-WT, G-R312C, and G-R312H were previously observed *in vitro* (16) and our aim was to provide context behind altered interactions with F-actin *in vitro*.

**Figure 3:**
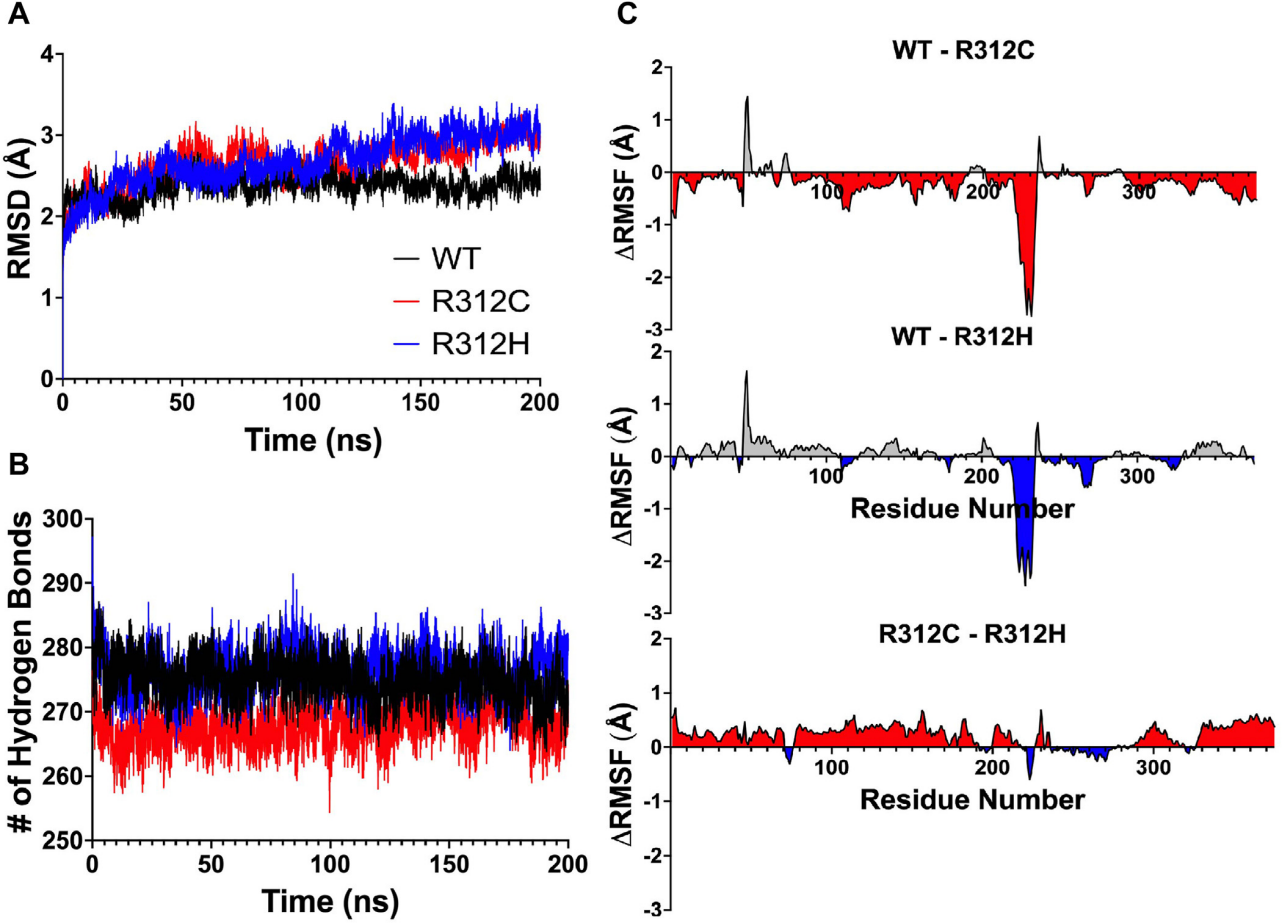
**Structural properties from F-actin simulations.** Chain C was isolated from each 5 protomer filament and analyzed. *A*, average root mean square deviation (RMSD) plot (N = 3 where N is an individual 200 ns replicate) displaying the average magnitude of changes in each structure over time, relative to the starting structure. WT (WT, colored *black*) plateaus around 50 ns, while R312C (*red*) and R312H (*blue*) diverge from WT and reach a plateau around 120 ns. *B*, the total number of protein-protein hydrogen bonds within the protomer over time (N = 3). R312C (*red*) displayed fewer bonds relative to WT (*black*) and R312H (*blue*), indicative of increased residue conformational freedom within the structure. The mean number of hydrogen bonds (±SD) for WT is 275 ± 7, R312C is 268 ± 8, and R312H is 277 ± 8. Differences in the average number of hydrogen bonds were found to be statistically significant (*p* < 0.0001) for all comparisons (WT *versus* R312C, WT *versus* R312H, and R312C *versus* R312H) following Kruskal–Wallis with Dunn’s Multiple Comparisons Tests. *C*, for a direct comparison of individual residue flexibilities, differences in average root mean square fluctuation (RMSF) are plotted (N = 3). RMSF values represent the average movement per residue over the entire simulation. RMSF values for each variant were subtracted from WT values, resulting in positive ΔRMSF representing more conformational freedom for residues in WT and negative ΔRMSF representing more residue flexibility in the variants. ΔRMSF for R312C is plotted in the *top* panel, and ΔRMSF for R312H in the *middle* panel. Both variants displayed increased flexibility in the Tm-bumper (residues 222–230) and decreased flexibility in the D-loop (residues 38–52) relative to WT. For comparing variants, the RMSF values for R312H were subtracted from R312C, demonstrating increased residue flexibility within R312C relative to R312H across most residues.

In G-actin systems, the G-R312C/H variants showed similar structural properties as G-WT (Fig. S1), in line with previous *in vitro* results (16). In F-actin systems, chain C was chosen for all analyses as it was the only F-actin protomer surrounded by other protomers (Fig. 1*C*). RMSD values, representing changes over time relative to the starting structure, were similar over 100 ns of the simulation before F-R312C/H diverged from F-WT (Fig. 3*A*). The largest differences in RMSD occurred in SD3 (Fig. S2*C*).

Fewer protein-protein hydrogen bonds were observed for F-R312C, suggestive of increased residue conformational freedom (Fig. 3*B*). Plotting Δroot mean square fluctuation (ΔRMSF) values shows that residues were more flexible across the F-R312C actin protomer (Fig. 3*C*). In both F-R312C and F-R312H systems, a helix directly above residue 312 containing residues 222 to 230 and referred to here as the ‘Tm-bumper’ due to its positioning near Tm-binding sites displayed substantial increases in flexibility (Fig. 3*C*). The DNaseI-binding loop (D-loop) displayed reduced flexibility in both F-R312C/H, particularly in residues 46 to 50 (Fig. 3*C*, Fig. S2*E*). Notable differences in RMSF values were also observed in select regions throughout F-actin structures (Fig. S2), including the hydrophobic plug containing residues 262 to 274 (Fig. S2*F*), a stretch of Tm binding residues from 318 to 338 (Fig. S2*G*), and the C-terminus from residues 350 to 375 (Fig. S2*H*). Differences in RMSF values were also calculated between F-R312C and F-R312H, indicating increased flexibility within F-R312C relative to F-R312H.

### Modeling suggests that R312C/H substitutions induce structural changes throughout actin

Actin structural changes were examined using cluster analyses, which identified commonly accessed conformations over the course of MD simulations. Analyses revealed similar structural changes in both the G- and F-actin states.

**Figure 4:**
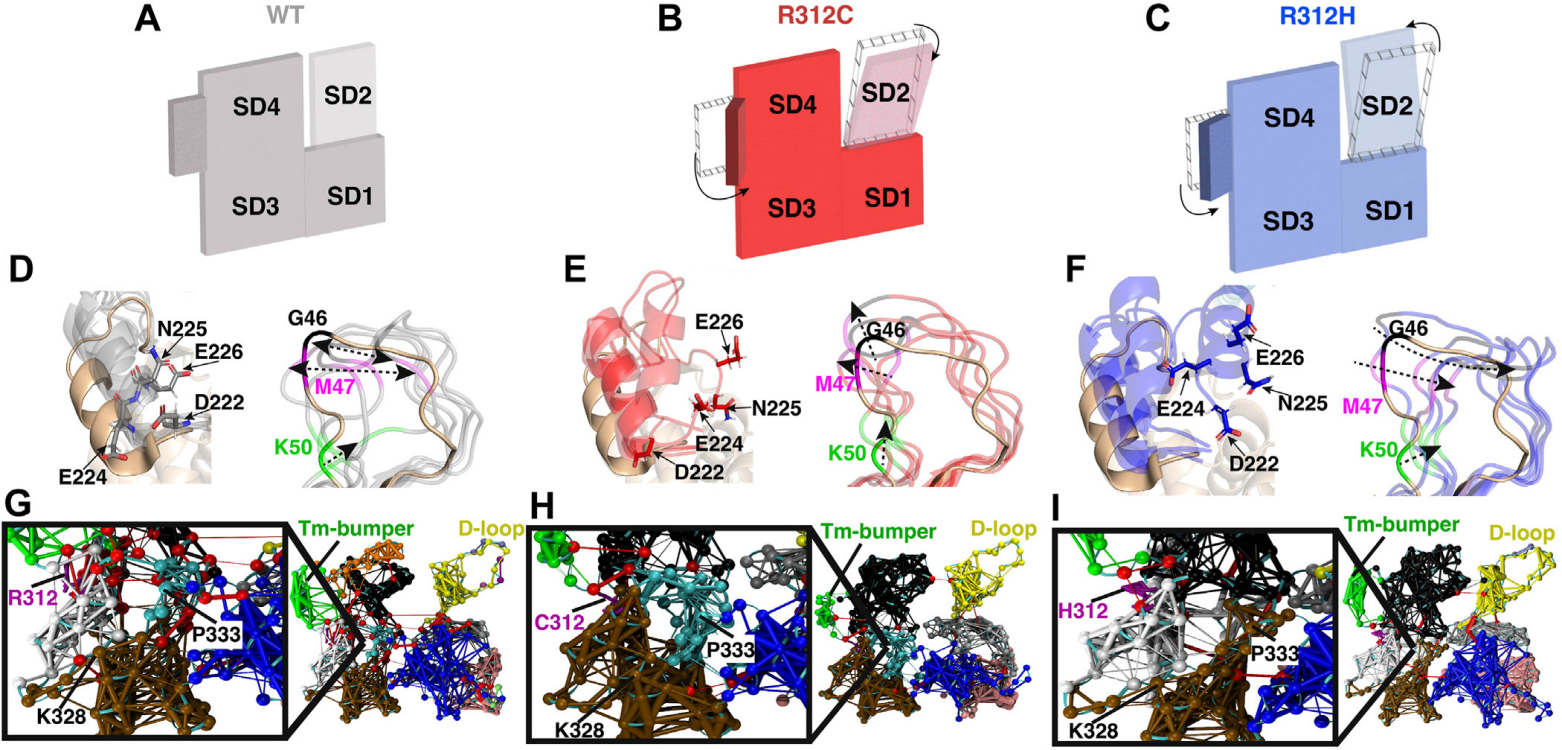
**Predicted structural changes from MD simulations.** The most common structures of chain C from F-actin were isolated and analyzed. *A*, a cartoon representation of the WT protomer, showing its relative stability across the four subdomains. *B*, a cartoon representation of the R312C protomer, showing a forward swing of the Tm-bumper (residues 222–230) on the outside of SDs three-fourths as well as a forward shift of the D-loop in SD2. *C*, a cartoon representation of the R312H protomer, showing a forward swing of the Tm-bumper, with less magnitude than in R312C, as well as a backwards shift of the D-loop in SD2. *D*, a structural superposition of the five most common WT conformations (colored *gray*) to the starting structure (colored *wheat*), with the Tm-bumper and D-loop shown. The Tm-bumper is shifted slightly forward relative to the starting structure, but all five clusters assumed a similar conformation. The D-loop, with residues G46 (*black*), M47 (*magenta*), and K50 (*green*) colored for reference, exhibits some flexibility by moving in all directions. *E*, a structural superposition of the five most common R312C conformations (colored *red*) to the starting structure (colored *wheat*). The Tm-bumper can shift forwards relative to WT clusters, shown by the position of residues D222/E224/N225/E226. The D-loop exhibits a shift ‘forward’ away from the central filament axis, most notable in residues G46 and M47. *F*, a structural superposition of the five most common R312H conformations (colored *blue*) to the starting structure (colored *wheat*). The Tm-bumper can shift far forward relative to WT clusters, as shown by the position of residues D222/E224/N225/E226. The D-loop exhibits a shift ‘backward’ towards the central filament axis, most notable in residues G46 and M47. *G*, dynamic network analysis of the WT protomer. Correlated movements were identified between residues over the course of all three 200 ns replicates, which were grouped together into individually colored communities. The thickness of connections between residues represents the strength of the connection. *Red* colored residues and connections represent critical nodes, important connections between communities. The protein backbone is shown as a ribbon, colored *cyan*. In the sub-panel, a close-up of SD3 is shown with residue R312 colored *pink* and its side chain shown, and residues K328/P333 identified for reference. *H*, dynamic network analysis of the R312C protomer. In the sub-panel, a close-up of SD3 is shown with residue C312 colored *pink* and its side chain shown, and residues K328/P333 identified for reference. *I*, dynamic network analysis of the R312H protomer. In the sub-panel, a close-up of SD3 is shown with residue H312 colored *pink* and its side chain shown, and residues K328/P333 identified for reference. Critical nodes are reduced in both R312C/H variants, indicating more isolated communities. The number and sizes of communities are altered throughout the structure, including the *cyan* colored community around P333, the *black* colored SD4, and the *yellow* colored D-loop, displaying how changes around residue 312 propagate throughout the entire structure and result in the observed D-loop shifts.

The five largest clusters for chain C for each F-actin system (Fig. 4) and each G-actin system (Fig. S3) revealed large-scale structural changes in the Tm-bumper helix (residues 222–230) in SD4. WT clusters showed a narrow range of motion for the Tm bumper (Fig. 4, *A–D*, Fig. S3*A*) due to the presence of stabilizing interactions between residues R312, A220, and E259 (Figs. S3*B* and S4, *A* and *B*). In the R312C variant, the loss of stabilizing interactions allowed C312 to form favorable S-H/π interactions with the side chain of F262 in four of the five clusters analyzed in both G- and F-actin systems (Figs. S3*D* and S4, *C* and *D*), resulting in a large swinging motion of the Tm-bumper (Fig. 4, *B–E*, Fig. S3*C*) stabilized by residue F223:C312 interactions (Fig. S4*C*). Stabilizing interactions are often present but weakened in the R312H variant. All clusters in both the G- and F-actin states showed H312 associating with the side chains of F262 and, geometry depending, E316 (Figs. S3*F* and S4, *E–F*), increasing conformational freedom for the 218 to 221 loop and causing shifts of the Tm bumper (Fig. 4, *C–F*, Fig. S3*E*). Both R312H and C showed some forward shifts in the Tm-bumper, with R312C (11% of the simulation) being more prevalent than R312H (4% of the simulation) (Table S3).

Consistent shifts in the D-loop were observed in F-R312C and F-R312H clusters (Fig. 4). D-loop residues 43 to 50 tended to shift ‘forward’ away from the central filament axis in most F-R312C clusters (Fig. 4, *B–E*), resembling what is referred to as the ‘closed’ D-loop conformation associated with flexible ADP actin filaments. Conversely, F-R312H clusters indicated a ‘backward’ shift toward the central filament axis (Fig. 4, *C–F*), resembling the ‘open’ D-loop conformation associated with more rigid ATP and ADP-Pi actin filaments. Conformational shifts in the D-loop and Tm bumper were supported by principal components analysis (PCA) by projecting extreme motions onto actin’s structure (Fig. S5).

The propagation of structural changes was supported by dynamic network analysis, which examines changes to regions of correlated movement, referred to as communities. In both variants, the number of critical nodes (connections between different communities) were reduced (Fig. 4, *G–I*). Fewer connections between the Tm bumper and SD4 were observed, confirming that this helix acts independently and moves with greater magnitude in F-R312C/H simulations. Further, both variants saw changes to communities in SDs 1 and 2 across the protomer. In the vicinity of residue 312, the 305 to 308 and 332 to 336 loops, which exhibited changes to residue flexibility (Fig. S2*G*), act as a small independent network in F-WT simulations (Fig. 4*G*) but is expanded around the nucleotide cleft in F-R312C simulations (Fig. 4*H*) and eliminated in F-R312H (Fig. 4*I*).

G-actin simulations revealed structural changes in similar regions as F-actin. Cluster analyses revealed large shifts in the Tm bumper for G-R312C (Fig. S3), with smaller shifts observed in G-R312H and reduced network connections between this helix and SD4 (Fig. S1, *G*, *J* and *M*). Large changes to SD1 and 2 communities were also observed, demonstrating consistency between structural states.

Consistent structural shifts are predicted at the Tm bumper, D-loop, and within SD3 in the vicinity of residue 312. These regions all lie within the binding sites of ABPs myosin, Tm, Tn, and cMyBP-C, which had altered interactions predicted by our *in vitro* results. The direct effect of actin structural changes due to R312C/H substitutions on ABP binding was therefore examined.

### Interaction energy between actin and ABPs tropomyosin, troponin, and myosin

To assess impacts on ABP binding, interaction energies between each F-actin system and myosin, troponin, and tropomyosin were measured. The 10 most common conformations adopted for F-actin’s chain C, representing 50%+ of total simulation time (Table S3), were isolated from a cluster analysis and used to build actin filaments. Individual ABPs were docked to these filaments using successive minimizations in a previously established method (17, 20, 21, 22).

**Figure 5:**
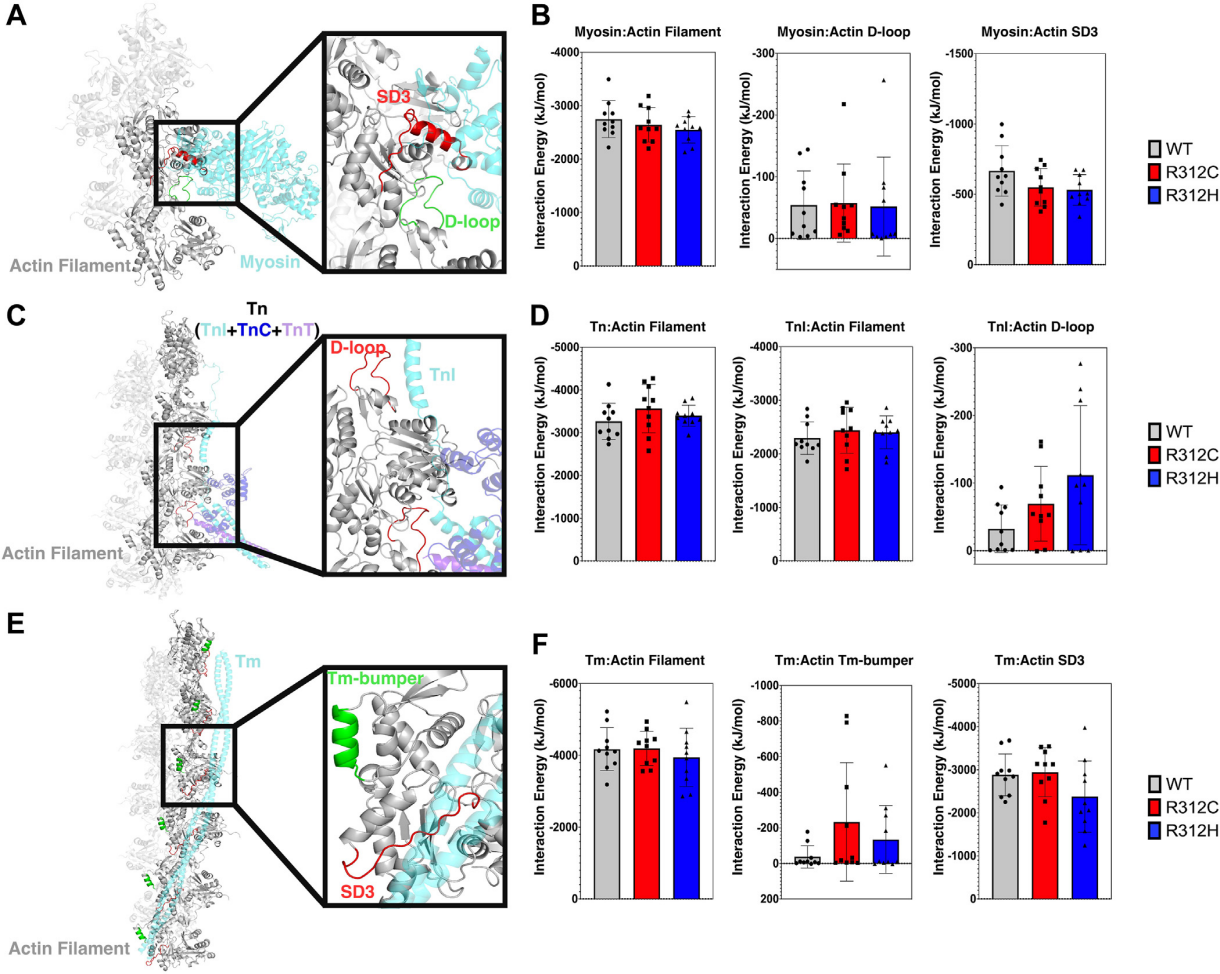
**Actin interaction energies.** Interaction energies were calculated between actin filaments conformations and docked ABPs myosin, troponin, and tropomyosin. For all plots, N = 10 where N is an actin structural cluster. All energy values are plotted as individual points, with bar graphs representing the average and error bars showing SD. *A*, myosin (colored *cyan*) in the open state was isolated from PDB 8EFI and docked to a 5 protomer filament through successive energy minimizations. Interaction energies were calculated between myosin and the two interacting chains (colored *dark gray*), the interacting SD3 in one protomer comprising residues 326 to 351 (colored *red*), and the interacting D-loop from one protomer (residues 38–53). *B*, average myosin:actin interaction energies were plotted for WT (*gray*), R312C (*red*), and R312H (*blue*). Average energy values are listed in Table S4. *C*, the troponin complex (TnI colored *cyan*, TnC colored *dark blue*, TnT colored *purple*) in the blocked state was isolated from PDB 7UTL was docked to an 8 protomer filament through successive energy minimizations. Interaction energies were calculated between the entire troponin complex and the 4 interacting actin protomers (colored *dark grey*). Energies were also calculated between TnI and the 4 interacting actin protomers as well as the two interacting D-loops (colored *red*) comprising residues 38 to 53. *D*, average troponin:actin interaction energies were plotted for WT (*gray*), R312C (*red*), and R312H (*blue*). Average energy values are listed in Table S5. *E*, tropomyosin in the blocked state isolated from PDB 7UTL was docked to an 11 protomer filament through successive energy minimizations. Interaction energies were calculated between the tropomyosin and the six interacting actin protomers (colored *dark grey*). Energies were also calculated between tropomyosin and all interacting Tm-bumpers (colored *green*, residues 222–230) as well as all interacting SD3 residues 321 to 335. *F*, average tropomyosin:actin interaction energies were plotted for WT (*gray*), R312C (*red*), and R312H (*blue*). Average energy values are listed in Table S6.

Interaction energies (sum of the short range Coulombic and Lennard-Jones potentials) were calculated between actin and myosin as well as Tm and Tn in the blocked state (Fig. 5, Tables S4–S6, for an overview see Table S2). Average interaction energies for R312C were more favorable for the myosin:D-loop, Tn:F-actin, TnI:F-actin, TnI:D-loop, and Tm:Tm-bumper interactions, relative to WT. Average interaction energies for R312H were more favorable for the Tm:Tm-bumper, Tn:F-actin, TnI:F-actin, and TnI:D-loop interactions, but less favorable for myosin:F-actin and myosin:actin SD3 as well as Tm:F-actin and Tm:SD3 interactions, relative to WT. As the main structural differences between R312C and R312H were opposing D-loop shifts, average D-loop:ABP distances were calculated (Fig. S8*A*). Strong correlations were observed between the D-loop proximity and interaction energies for the myosin:F-actin (Fig. S8*B*, r = 0.4641 and *p* = 0.0098), myosin:D-loop (Fig. S8*C*, r = 0.6848 and *p* < 0.0001), and TnI:D-loop (Fig. S8*E*, r = 0.7000 and *p* < 0.0001) interactions. Interaction energies were also calculated between actin and Tm as well as Tn in the closed state (Table S6). For Tm relative to the blocked state, overall interaction energies were less favorable for WT but more favorable for R312C/H.

## Discussion

HCM and DCM are thought to occur from hyperactivity and hypoactivity in the sarcomere, respectively, due to changes in contractile protein interactions. Much like in other muscle types, changes in cardiac sarcomere contraction affects the development of heart musculature: hyperactivity and increased contraction (HCM) causes a thickening, hypoactivity and decreased contraction (DCM) causes a thinning of the musculature. Our earlier work suggests that R312H (found in a patient with DCM) is hyperactive *in vitro*, suggestive of increased sarcomere contraction and HCM, while both R312C/H variants also displayed decreased calcium sensitivity (16), suggesting hypoactivity and DCM. Here, we utilized *in vitro* and *in silico* methods to assess the R312C/H variants and identify mechanisms driving discrepancies in their activity. In addition, we sought to clarify differentiating factors between HCM- and DCM-causing variants, as the details of *how* sarcomere protein interactions are affected in one disease *versus* the other remains unknown.

### Changes to tropomyosin binding in R312C/H variants

Previous assays performed with the R312-ACTC variants have all displayed an impact on the regulation of contraction (16, 23, 24, 25, 26). As Tm is a main regulator of actomyosin activity, changes to this interaction could alter sarcomere activity, resulting in CM. Arginine 312 lies in a critical region of actin for Tm interactions (Fig. 1*E*), neighboring Tm-binding D311 (16, 27, 28) and in the vicinity of Tm-binding K326, K328, and A331 which have been correlated with CM (29, 30, 31). We therefore speculated that destabilization of this region due to amino acid substitutions at R312 would reduce energetics for Tm binding, altering the regulation of contraction in CM.

*In vitro*, we observed no differences in the equilibrium binding of Tm (Fig. 2, *A* and *B*, Table 1). Under real-time kinetic conditions, though complete saturation was observed after 60 s, the rates of Tm elongation in the R312C/H variants were reduced relative to WTRec (Fig. 2*E*, Table 1). As the equilibrium co-sedimentation assay occurs over a 60-min period, the complete saturation after 60 s under kinetic conditions explains why no differences in the equilibrium binding were observed.

*In silico* modeling suggests that less favorable interactions with key Tm-binding residues in SD3 (residues 320–334) in the R312C/H variants (Fig. 5*F*), coupled with interference from a highly mobile Tm-bumper (residues 222–230) in both variants (Fig. S6, *A*, *D*, *G* and *J*), might disrupt the rate of Tm binding and elongation. R312C also exhibited the lowest elongation rate (Fig. 2*E*), coinciding with more frequent forward shifts in the Tm-bumper relative to R312H. Therefore, there is evidence that structural shifts resulting from R312C/H substitutions alter interactions between actin and Tm in the blocked state.

### Altered regulation of the blocked state in R312C/H variants

In the absence of troponin, Tm maintains an equilibrium A-state that inhibits myosin binding and activity (17), something that would be affected by altered Tm binding. *In vitro*, the R312C/H variants exhibited reduced Tm-inhibition of myosin in the absence of troponin (Fig. 2*F*), as well as hyperactivity under low calcium conditions in the presence of troponin (16). Taken together, myosin can generate filament motility in both R312C/H variants under conditions where its binding sites are blocked in WTRec actin. Therefore, the localized A-state of Tm in the absence of Tn, as well as its blocked (B-) state in the presence of Tn under low calcium conditions, might be destabilized in R312C/H variants. Destabilized A-/B-states would increase the exposure of myosin-binding sites, reducing Tm’s inhibitory activity and increasing actomyosin activity relative to WTRec.

Destabilization of the A- and B-states for the R312H variant is supported by less favorable interactions between Tm and key stabilizing residues in actin SD3 (residues 320–328) relative to WT and R312C (Fig. 5*F*, Table S6), which displayed greater hyperactivity than R312C under low calcium conditions *in vitro*. Further, both R312C/H variants display substantially more favorable interaction energies in the closed (C-) state conformation relative to the B-state (Table S6), potentially driving transitions away from the B-state conformation. In addition, troponin I (TnI) binds directly to the D-loop (residues 38–52) and directs the accessibility of myosin-binding sites. Structural changes in the vicinity of residue 312 propagate to the D-loop *in silico*, with D-loop positioning correlating with TnI interaction favorability (Fig. S8, *D–F*). R312C/H filaments might therefore alter TnI positioning and stability, further altering the accessibility of myosin-binding sites.

Therefore, we propose that structural changes in the vicinity of residue 312 induce shifts in the conformation and properties of nearby residues (320–328), disrupting actin:Tm interactions. Destabilized Tm might shift away from the blocked state *via* interactions with a highly mobile Tm-bumper that pulls Tm towards the more favorable closed state or repositioning by TnI which has been destabilized due to D-loop shifts. Destabilized regulatory proteins would increase the exposure of myosin-binding sites within R312C/H variants, leading to hyperactivity under inhibitory conditions that increases overall sarcomere contractile activity. Impaired regulation of contractile activity is suggestive of increased musculature in the heart and HCM.

### Structural changes might explain decreased calcium sensitivity

While R312C/H variants exhibit hyperactivity under low calcium conditions characteristic of HCM, both variants also display decreased calcium sensitivity characteristic of DCM (16). In principle, destabilized Tm/Tn in the blocked state as we have suggested should result in reduced energy barriers between Tm-binding states, allowing for easier transitions to the open state and increasing calcium sensitivity. Structural rearrangements of the Tm-bumper, however, can result in steric clashes with Tm’s open state conformation (Fig. S6, *C*, *F*, *I* and *L*). Therefore, while closed state accessibility might be improved in R312C/H, if Tm’s subsequent closed to open state transition occurs at a lower frequency due to Tm-bumper positioning, average myosin activity would be reduced relative to WT, leading to the observed rightward pCa shift *in vitro*.

### R312C/H alters the actomyosin interaction

Substitutions at residue 312 appear to alter sarcomere contractile activity through two mechanisms: impaired regulation of actomyosin interactions by Tm/Tn and direct effects on the actomyosin interaction. Previously, we observed increased maximal IVM velocities (V_max_) and actomyosin ATPase activity for R312C/H variants in the absence of Tm and Tn (16).

Structural shifts and changes in flexibility are observed in the vicinity of residue 312 within SD3 residues 318 to 328 and 336 to 355 (Fig. S2), which constitutes a major section of the myosin-binding site (28, 32, 33, 34). Further, D-loop shifts are observed *in silico* which might play a role in regulating initial myosin binding (34). Overall, myosin binding to R312H appears to be less favorable due to shifts in the D-loop and myosin-binding residues in SD3 (Fig. 5*B*). A reduction in the strength of the actomyosin interaction could increase the rate of detachment and lead to greater V_max_ while reducing force output (16), as supported by recent models (35). R312C displays more favorable myosin:D-loop interaction energies due to closer proximity, possibly reflective of decreased K_M_ in unregulated IVM assays (16). Tighter binding between R312C’s D-loop conformation and myosin is supported by previous findings of myosin’s strong binding selecting for D-loop conformations that resemble those seen in R312C (36), potentially increasing the duty ratio and overall force output (35, 37). The suggestion that D-loop shifts observed *in silico* impact the favorability of actomyosin interactions is supported by strong correlations between the average myosin:D-loop distance and myosin interaction energies (Fig. S8, *B* and *C*).

### Regulation by C0C2 might be altered in R312C/H

Regulation of contraction does not occur solely through Tm/Tn binding. The C0C2 domains of myosin binding protein C (cMyBP-C) also participate in thin filament regulation by shifting Tm positioning and increasing force production (6, 7, 8, 9), altering thin filament activity *in vitro*. We show that, relative to WT, R312C/H variants continued to exhibit increased activity under low calcium conditions, R312H displayed hyperactivity at all pCa levels, and both variants became more sensitive to calcium (Fig. 2*G*).

C0C2 binds to the interprotomer interface and the Tm-bumper. Therefore, predicted shifts in the D-loop and Tm-bumper could influence C0C2 binding and subsequent regulation. For example, it is possible that C0C2 binding to the Tm-bumper prevents the forward shifts that reduce access to the open state. If Tm’s blocked state is destabilized in R312C/H variants and the open state is accessible, Tm might shift into the open state more frequently and increase calcium sensitivity relative to WT.

Further, C0C2 binds directly to D-loop residues 46 to 49, which move away from C0C2 in F-R312H *in silico*. We postulate that such shifts might reduce the favorability of this interaction, supported by previous studies demonstrating impaired C0C2 binding with R312H filaments (38). We also know that the C1C2 domains reside close to the myosin-binding site (39, 40, 41), changing the actomyosin interaction to reduce velocities *in vitro* (19). It is therefore possible that impaired binding to R312H reduces C0C2’s velocity dampening functions, leading to the observed hyperactivity at all pCa levels and contributing to sarcomere hyperactivity. Further studies into C0C2 binding dynamics, regulation, and structure, however, are still required to confirm these mechanisms.

### Study limitations

Detailed characterization of R312C and R312H has revealed changes to underlying mechanisms that might cause altered sarcomere contraction *in vivo*. Our results are also supported by similar studies characterizing actin variants M305L (17) and A295S (42) *in vitro* and *in silico*, which observed altered calcium sensitivity, Tm-inhibition, as well as similar structural changes throughout actin including Tm-binding residues and the D-loop. There are, however, some limitations that prevent us from developing a complete picture describing the mechanisms leading to disease in these variants. For example, the ACTC proteins utilized for *in vitro* characterization were expressed in baculovirus, which may lack some posttranslational processing that is present in mammals; however, any differences are controlled through direct comparison between baculovirus-expressed ACTC proteins. Further, there are structural limitations preventing the calculation of complete energy landscapes across the entirety of an actin filament’s surface that would visualize shifts in highly favorable positions as has been done previously (17, 20, 21, 22). Namely, shifts in the Tm-bumper sterically interfere with Tm’s open state positioning and come into close proximity to Tm’s closed and blocked states, which would result in unresolvable and infinitely large forces during protein docking. In addition, docking could not be completed for C0C2 as no current structure of C0C2 bound to actin exists. As a result, we must infer changes to the actin:C0C2 interaction from the available data. Finally, high-resolution electron microscopy is required to support the *in silico* modeling presented here, which can be used to identify variant-specific conformational changes as well as the impacts on actin:ABP interactions (34, 43).

## Conclusions

HCM- and DCM-causing actin variants are typically associated with hyperactivity and hypoactivity within the sarcomere, respectively. Actin variants, however, do not appear to act as a monolithic group. Previously, we characterized two actin variants with substitutions at residue 312 that have been connected to two forms of cardiomyopathy, R312C (HCM) and R312H (DCM). Both variants displayed the same pattern of hyperactivity and hypoactivity under different conditions, clouding the underlying mechanisms that differentiate these diseases. We therefore hypothesized that a gradient of factors such as actomyosin force output, activity under low calcium conditions, and altered regulation by proteins such as cMyBP-C determine the characteristics of cardiomyopathy, rather than uniform shifts in activity. Here, further *in vitro* characterization supported our previous findings (16) identified altered regulatory abilities of tropomyosin and cMyBP-C, while *in silico* modeling identified structural changes that might drive the observed *in vitro* behavior.

R312C was found in a patient with HCM (4), typically characterized by sarcomere hyperactivity. *In silico*, movement of the Tm-bumper (residues 222–230) might reduce the frequency of tropomyosin’s shift to the open state in the presence of calcium, reducing calcium sensitivity as seen *in vitro* which is suggestive of hypoactivity. Related structural shifts in actin’s D-loop, however, might increase the strength of actomyosin interactions, increasing actomyosin force output as observed previously. Further, shifts in actin SD3 might destabilize actin:Tm interactions in the blocked state and drive a transition to a more energetically favorable closed state. Increased actomyosin force output coupled with increased myosin-binding site exposure through reduced Tm-inhibition could result in hyperactivity characteristic of HCM.

R312H has been found in patients with DCM, which is typically characterized by sarcomere hypoactivity. While R312H exhibited decreased actomyosin force output and calcium sensitivity *in vitro*, it exhibited hyperactivity under most conditions that is characteristic of HCM. We postulate that such hyperactivity in R312H, particularly under low calcium ‘relaxing’ conditions, prevents sarcomere relaxation, resulting in deterioration and subsequent dilation of the heart musculature (DCM) either directly or from gradual progression to DCM from HCM (11).

While both variants displayed similar *in vitro* characteristics and *in silico* structural shifts, variant-specific D-loop shifts might play an important functional role by differentiating myosin attachment and detachment kinetics, leading to variable sarcomere contraction and HCM-related and DCM-related changes for R312C and R312H, respectively. Behavior contradicting traditional characterizations of HCM and DCM has been observed previously, for example in the HCM-causing myosin R403Q variant which reduces motor activity (15). The finding that cardiomyopathy-causing variants can present both hyperactivity and hypoactivity in different contexts requires further investigation to understand the specific mechanisms altering sarcomere contraction. It is clear, however, that a multitude of factors from actomyosin force output to the regulatory functions of several ABPs must be considered when characterizing protein variants connected to cardiomyopathy, as simple definitions of hyperactivity for HCM and hypoactivity for DCM present an incomplete picture.

The data presented here suggest that R312C/H actin variants experience actin structural shifts that might impact interactions with Tm, Tn, myosin, and C0C2, resulting in altered sarcomere regulation and contraction through similar mechanisms that differentiate in their magnitude. Additional studies, however, such as the generation of high-resolution R312C/H actin structures are still needed to confirm the mechanisms presented here. Developing a complete understanding of actin structural changes within sarcomere interactions can form the foundation for in-depth predictive models of altered sarcomere activity in HCM *versus* DCM, guiding clinical treatments for cardiomyopathy and improving clinical outcomes.

## Experimental procedures

### Reagents

All reagents, unless otherwise specified, were purchased from Fisher Scientific or Sigma-Aldrich. HiTrap DEAE and Sephadex G25 Desalting fast flow columns (Cytiva Life Sciences) and Ni-NTA Super-flow columns (Qiagen) were used for recombinant ACTC purification. IMAX Serum Free Media was obtained from Wisent Bioproductions and Penicillin/Streptomycin mix was obtained from Gibco Life Technologies. ATP used in recombinant ACTC purification was obtained from Bio Basic Inc. Equipment used also included an Optima Max E Ultracentrifuge (Beckman Coulter), and 100× TIRF objective and Nikon Eclipse Ti2 microscope (Nikon).

### Protein purification

All recombinant ACTC proteins were produced using a baculovirus expression system. The recombinant ACTC proteins were expressed in Sf9 insect cells infected with recombinant baculoviruses containing the ACTC1 variant of interest and purified with hexa-His-tagged gelsolin segments 4 to 6 (G4-6) as described previously (44). Tropomyosin and troponin were purified from bovine cardiac ether powder as outlined previously (45, 46). The myosin utilized for the *in vitro* motility was purified from rabbit *soleus* muscle which was then cleaved into heavy mero-myosin (HMM) using a-chymotrypsin as previously outlined (47). Bovine cardiac myosin HMM was purified from left ventricular tissue for the TIRF-based binding assay using the same method based on the availability of tissue (47, 48). The *soleus* muscle of rabbits has a similar ratio of fast and slow muscle fibers to that found in cardiac tissue, with greater than 90% slow myosin and less than 10% fast myosin (49, 50). This similarity in fiber type distribution makes rabbit soleus muscle a suitable model for studying muscle proteins relevant to heart function. Recombinant C0C2 was purified using *Escherichia coli* (*E. coli*) Rosetta cell expression with subsequent nickel affinity chromatography as described (19), but with slight modifications. The cells were lysed using a French press high pressure system and the clarified lysate was flowed on to a Ni-NTA fast flow column and purified using an FPLC. The presence of the proteins of interest were confirmed using SDS-PAGE analysis.

### Tropomyosin Co-sedimentation and motility changes

Tropomyosin equilibrium binding was assessed with a co-sedimentation assay described previously with slight modifications (51). Tropomyosin was purified from bovine ventricular ether powder (46). F-actin (7 μM) was combined with 0.1 μM to 2 μM tropomyosin in (100 mM KCl, 5 mM MgCl_2_, and 20 mM Tris pH 8) for 1 h at 25˚C. Following incubation, the actin-tropomyosin mixture was centrifuged at 100,000 rpm for 20 min in a TLA100 rotor, and samples of the pellet were run on an SDS-PAGE gel. The concentration of the Tm in the pellet was determined with densitometry analysis using ImageJ (National Institutes of Health) and plotted against the total Tm added in the respective sample. Data was normalized from 0 to 100 where 100 percent represented the maximum Tm level in the respective assay. Analysis of the binding curves was performed in GraphPad Prism 10 by Dotmatics (San Diego) fitting the data to a specific binding with Hill slope function.

Thin filaments composed of actin, Tn, and Tm can be characterized through many assays, demonstrating the functional impacts of amino acid substitutions. Thin filament characterization assays include variations of the IVM assay, where fluorescently labelled actin filaments in the presence or absence of regulating proteins Tn and Tm move across a surface of active myosin heads, with filament velocity providing insights into protein activity and interactions. Changes in F-actin motility due to inhibition by Tm in the absence of Tn were assessed using an IVM assay, with data collection as described previously (16, 52). Three independent purifications were tested for each ACTC-variant of interest. Three videos for each assay condition (No Tm and 3 μM Tm, in a motility buffer containing 4 mM MgCl_2_, 1 mM EGTA pH 8, 25 mM Imidazole pH 8, 25 mM KCl, 0.432 mg/ml Glucose Oxidase, 0.072 mg/ml Catalase, and 2 mM ATP pH 8) were captured for each preparation. The HMM concentration used was 0.5 mg/ml mixed in 1:1 fashion with 0.1 mg/ml BSA (4 mM MgCl_2_, 1 mM EGTA pH 8, 25 mM Imidazole pH 8, 25 mM KCl) to a final concentration of 0.25 mg/ml. This ratio of proteins ensured spacing in between the myosin proteins, to simulate a low-density myosin concentration. Within each video, the movement of 10 filaments were tracked manually in ImageJ over 10 consecutive video frames, determining velocities from distance travelled in that time frame. The velocities of the 10 filaments from each video were entered into GraphPad Prism 10 (N = 90), averaged, and displayed as a violin plot.

### TIRF-based tropomyosin binding

To assess the kinetic binding of Tm to F-actin, a TIRF-based Tm binding assay was performed as described previously, with slight modifications (53). The tropomyosin purified from bovine ventricular ether powder was covalently modified on Cys-190 with Alexa-488 maleimide from Fisher Scientific. Labelled Tm was further purified with washing buffer (10 mM Tris pH 7.5, 0.01% NaN_3_) through a Sephadex G25 desalting column to remove excess fluorophore. The efficiency of fluorophore binding was determined with a spectrophotometer (Direct Detect Spectrophotometer, MilliporeSigma). The absorbance at 493 nm (Alexa 488 maleimide peak absorbance) and 280 nm (Tm peak absorbance) gave a ratio of roughly 1.4:1 for the ratio of Alexa-488 maleimide to tropomyosin, similar to the 1.2 fluorophore to Tm ratio from previous literature (53). Tropomyosin was added to an IVM flow cell with a 30 mm × 22 mm glass coverslip (Fisher Scientific) coated in nitrocellulose attached to a custom aluminum microscope slide with a hole cut in the center, allowing for direct access between the TIRF objective and the glass coverslip. Bovine ventricular HMM (0.5 mg/ml) was added to the flow cell, washed with BSA buffer (0.1 mg/ml), followed by the ACTC variant of interest labelled with rhodamine phalloidin. Actin was visualized at 560 nm and focused with the 100× TIRF objective. The laser was adjusted to the 488 nm excitation wavelength, and Alexa-Tm was added directly while recording binding at 10 frames per second. Videos were exported as avi files and fluorescence intensity was observed along the length of an entire actin filament.

### cMyBPC (C0C2) IVM assay

As the presence of C0C2 influences calcium sensitivity, F-actin velocity, and constitutive thin filament activity *in vitro*, an IVM assay with the addition of C0C2 to a constant 0.25 mg/ml HMM was performed using methods similar to our previous regulated IVM assays (16). When adding regulatory proteins, 1 μM of C0C2 was also added to the 3 μM Tn/Tm mix; similar concentrations to previous IVM assays which assessed the impact of C0C2 on regulated thin filaments (19). In addition, troponin used for the Tn/Tm complex was purified from bovine ventricular ether powder as performed previously (45). The pCa levels ranged from 10-4.5 free calcium balanced using an online calculator based on literature values (54). The velocities of 10 filaments were assessed per video, with 3 videos taken per distinct ACTC purification, and 3 separate purifications per ACTC variant (N = 90). All velocities were compiled into GraphPad Prism 10.

### Generation of actin structural models

Initial structures of human alpha-cardiac actin (ACTC) were generated with MODELLER software (55, 56) using WT, R312C, and R312H sequences. For monomeric G-actin, the crystal structure of bovine beta-actin (PDB 2BTF) was used as a reference. For F-actin, a single protomer was isolated from a 5 protomer filament of F-actin in the Mg^2+^ ADP-Pi state (PDB 8A2S) and used as a reference structure. Models were assessed with the MODELLER objective function and discrete optimized protein energy scores to determine which of the 10 generated structures would be used for analysis. RMSD scores between the generated models and reference structures were 0.284 Å (G-actin) and 0.169 Å (F-actin) for WT, 0.285 Å (G-actin) and 0.203 Å (F-actin) for R312C, and 0.221 Å (G-actin) and 0.174 Å (F-actin) for R312H. Methylated histidine 73, Mg^2+^, ATP (G-actin), and ADP + Pi (F-actin) were added to each structure in PyMOL (The PyMOL Molecular Graphics System, Version2.0 Schrödinger, LLC) *via* structural superposition to the reference structures.

F-actin models comprising five actin protomers were then built from the MODELLER generated actin protomer, using structural superpositions in PyMOL to the 8A2S reference structure.

### MD simulations

MD simulations were conducted for all systems using the charmm36 forcefield (57) in GROMACS (58) through the Digital Research Alliance of Canada’s Graham computing cluster, located at the University of Waterloo.

All systems were fully solvated using the TIP3P explicit water model (59), with Na^+^ and Cl^-^ counter ions to neutralize net charge, and proteins were centered within a cubic box at a minimum distance of 1 Å from the edge of the box. Periodic boundary conditions were applied in all directions and the GROMACS recommended CHARMM36 parameters were used. Systems were minimized using steepest descent with a step size of 0.05 until the maximum force was less than 1000 kJ mol^-1^ nm^-1^. Minimized systems underwent 100 ps isothermal-isobaric equilibration at 310K and 1 bar. Production run 200 ns MD simulations for both G-actin and F-actin were conducted in triplicate (for an overview of systems studied, see Table S1) with 2 fs timesteps using the Berendsen thermostat and Parrinello-Rahman barostat (60) to maintain system temperature and pressure. Bonds were constrained with the LINCS algorithm (61). The particle-mesh Ewald method (62) was used for long-range electrostatics, while short-range electrostatics and van der Waal’s forces were cutoff at 1.2 nm. To maintain the overall filament organization, position restraints were applied to the backbone of F-actin residues 105, 162, and 157 of chains A, B, D, and E (see Fig. 1*C*).

### Analysis of MD simulations

Unless otherwise stated, all analyses were conducted using in house scripts. MD trajectories were first processed to account for jumps across periodic boundaries. For F-actin simulations, a single protomer (chain C, see Fig. 1*C*) was isolated and analyzed as it was the only protomer that maintained protein contacts in all directions and was thus representative of protomers within a larger filament. RMSD, RMSF, radius of gyration, hydrogen bonds, and bond distances were calculated from the individual replicate trajectories.

Processed replicate trajectories were then combined for each system (i.e. the three 200 ns replicates for WT F-actin were combined into a single 600 ns trajectory), and, in the case of F-actin systems, were processed to isolate the trajectory of chain C only before being subjected to further analyses. A cluster analysis was performed using the gromos algorithm (63) and a cutoff of 0.15 nm to identify common conformations sampled in the combined trajectories, from which the top five clusters by number of structures were isolated. Combined trajectories for each system were then used for dynamic network analysis to identify networks of correlated motions between C_α_ pairs, and the Girvan-Newman algorithm (64) was used to identify communities of correlated residues. Network communities were visualized with the NetworkView plugin (65) in VMD (66).

PCA was used to reduce the dimensionality of the data into dominant protein motions. PCA was conducted through the GROMACS *gmx covar* and *gmx anaeig* functions, which calculate and diagonalize the covariance matrix and determine eigenvectors before projecting a trajectory along designated eigenvectors. The two largest eigenvectors represented the two dominant motions over the course of the simulation and are hereafter referred to as principal components 1 and 2 (PC1 and PC2, respectively). First, PCA was conducted for the combined F-actin chain C trajectory of each system, with each system projected along their own eigenvectors to isolate PDB files visualizing the extreme motions that each system adopted over the course of the simulation. To allow a direct comparison of conformational freedom within each system, the combined 600 ns WT, R312C, and R312H trajectories were then combined into a single 1.8 μs trajectory (one for F-actin, one for G-actin). The 1.8 μs combined trajectories underwent PCA to determine dominant motions common across all individual systems. The individual combined trajectories (i.e. the 600 ns WT F-actin, 600 ns R312C F-actin *etc.*) were projected along the eigenvectors from their respective 1.8 μs combined trajectory, which were used to generate Gibbs free energy landscapes (FEL).

### Actin–ABP interaction energies

To determine the effects of R312C/H structural changes on the binding of different ABPs in the sarcomere, Tn, Tm, and myosin were docked to actin using previously established methods (17, 20, 21, 22). In short, a cluster analysis on combined trajectories for each F-actin system revealed the dominant conformations accessed over each simulation. The 10 largest clusters from each system were isolated, and filaments of varying length were built *via* structural superposition to actin filaments in three reference structures, PDB 7UTL, PDB 7UTI, and PDB 8EFI (see Table S2), representing thin filament structures in the Blocked, Closed, and Open states. Tn (chains U +V+Y) was isolated from PDBs 7UTL and 7UTI, Tm (chains i + j) were isolated from PDBs 7UTL and 7UTI, and myosin (chain M) was isolated from 8EFI.

All actin structures were minimized in the absence of any ABPs, using a combination of steepest descent and conjugate gradient until the maximum force was less than 50 kJ mol^-1^ nm^-1^. Separate actin-ABP structures were then built, with each ABP docked *via* structural superposition of the minimized filaments to reference structures 7UTL, 7UTI, and 8EFI in PyMOL. Tm C-terminal residues 254 to 284 were trimmed as per previous protocols (17, 20, 21, 22). Bound ABPs were then moved 5 Å away from the filament surface and minimized using a combination of steepest descent and conjugate gradient until the maximum force was less than 50 kJ mol^-1^ nm^-1^. The ABPs were then translated 0.5 Å closer to the actin filament and the system minimized, repeating this process until each ABP reached its original docking radius. A final minimization was conducted using conjugate gradient until the maximum force was less than 10 kJ mol^-1^ nm^-^1. The docking method employed allowed the resolution of poor actin-ABP contacts that might result from structural changes in actin due to the R312C/H substitutions. It should be noted that interaction energies were measured between actin and Tn/Tm in the Blocked and Closed states, and with myosin in the Open state. Interaction energies were not measured between actin and Tm in the Open state, due to steric clashes between multiple R312C/H clusters and the Tm Open state conformation resulting in infinitely large forces that are unresolvable through energy minimization alone. Further, interaction energies were not measured between actin and C0C2 due to the lack of a complete actin:C0C2 structure.

### Statistical analyses

Any reported statistical analyses were conducted in GraphPad Prism 10. All datasets were tested for normality with Shapiro–Wilk Tests. Normally distributed data was analyzed *via* one way ANOVA with *post hoc* Tukey Tests. Non-normally distributed data was analyzed *via* Kruskal–Wallis with Dunn’s Multiple Comparisons Tests.

## Data availability

All data for this publication are presented in the article and supporting information. Excel files containing raw data is available upon request from John F Dawson (jdawso01@uoguelph.ca).

## Supporting information

This article contains supporting information.

## Conflict of interest

The authors declare that they have no conflicts of interest with the contents of this article.
