# Supplementary material for: Duality in disease: How two amino acid substitutions at actin residue 312 result in opposing forms of cardiomyopathy
Source: J Biol Chem. 2024 Nov 5;300(12):107961. doi: 10.1016/j.jbc.2024.107961 (PMC11652881; doi:10.1016/j.jbc.2024.107961)
Supplement: Supplemental Figures S1–S8 [file mmc1.docx]

**
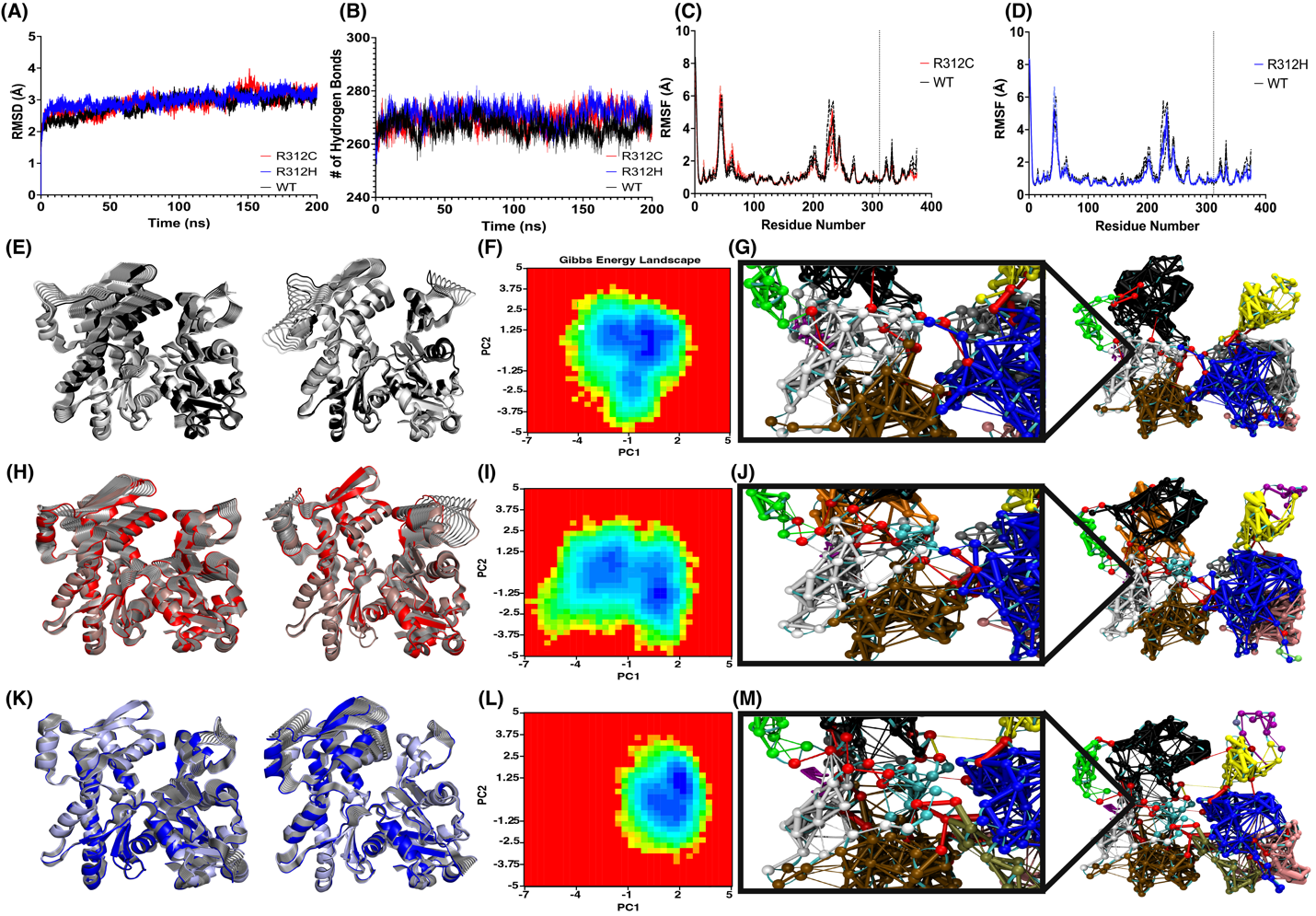
**

**Figure S1 G-actin *in silico* modelling. (A)** Average RMSD plots (N=3) for G-actin WT (black), R312C (red), and R312H (blue) showing similar magnitudes of changes for the entire monomer over the course of each simulation. **(B)** Average number of hydrogen bonds throughout the monomer (N=3) over the course of the entire simulation. Similar numbers of bonds were observed between WT, R312C, and R312H. **(C)** Average RMSF values (N=3) for R312C (red) and WT (black). Dotted lines represent SEM. **(D)** Average RMSF values (N=3) for R312H (blue) and WT (black). Dotted lines represent SEM. **(E)** A principal component analysis was conducted for each system to reduce the dimensionality of the data. The extreme motions from the two largest principal components (PC1 and PC2), representing the two main types of motion observed, were projected onto the monomeric structure. WT monomers exhibited the largest range of motion in SDs 2 and 4. **(F)** Gibbs free energy landscape (FEL) for the WT monomer along PC1 and PC2, calculated based on the number of simulation timepoints adopting coordinates along these two principal components. Red represents few timepoints accessed those coordinates and are therefore unfavourable conformations. Dark blue represents many timepoints accessed those coordinates, representing favourable conformations. **(G)** Dynamic network analysis for the WT monomer, with the side chain of R312 coloured magenta and shown in the sub-panel. **(H)** Projections of the extreme motions along PC1/PC2 onto the R312C monomer, showing large ranges of motion in SDS 2 and 4. **(I)** FEL for the R312C monomer along PC1 and PC2, showing a wider energy basin than WT indicative of a wider range of conformations accessed over the simulation. **(J)** Dynamic network analysis for the R312C monomer, with the side chain of C312 coloured magenta and shown in the sub-panel. The size and number of communities containing correlated residues are altered throughout the structure, with changes visible in the same regions as F-actin network analyses (**Figure 4**). For example, the cyan coloured network around residue P333 does not exist in WT, demonstrating that it acts independently in the variant, but operates as part of the larger SD3 communities in WT. **(K)** Projections of the extreme motions along PC1/PC2 onto the R312H monomer, showing reduced ranges of motion in SDS 2 and 4 relative to WT. **(L)** FEL for the R312H monomer along PC1 and PC2, showing a smaller energy basin than WT, indicative of a smaller range of conformations accessed over the simulation. **(M)** Dynamic network analysis for the R312H monomer, with the side chain of H312 coloured magenta and shown in the sub-panel. The size and number of communities containing correlated residues are altered throughout the structure, with changes visible in the same regions as F-actin network analyses (**Figure 4**). The independent cyan coloured network around residue P333 is expanded relative to R312C, demonstrating that this region has a greater independence than in WT. A magenta coloured community is also present in the D-loop, similar to R312C, which isn’t present in WT, suggesting that changes at residue 312 affect the makeup of SD2 which is supported by changes in its range of motion as shown by PCA projections.

**
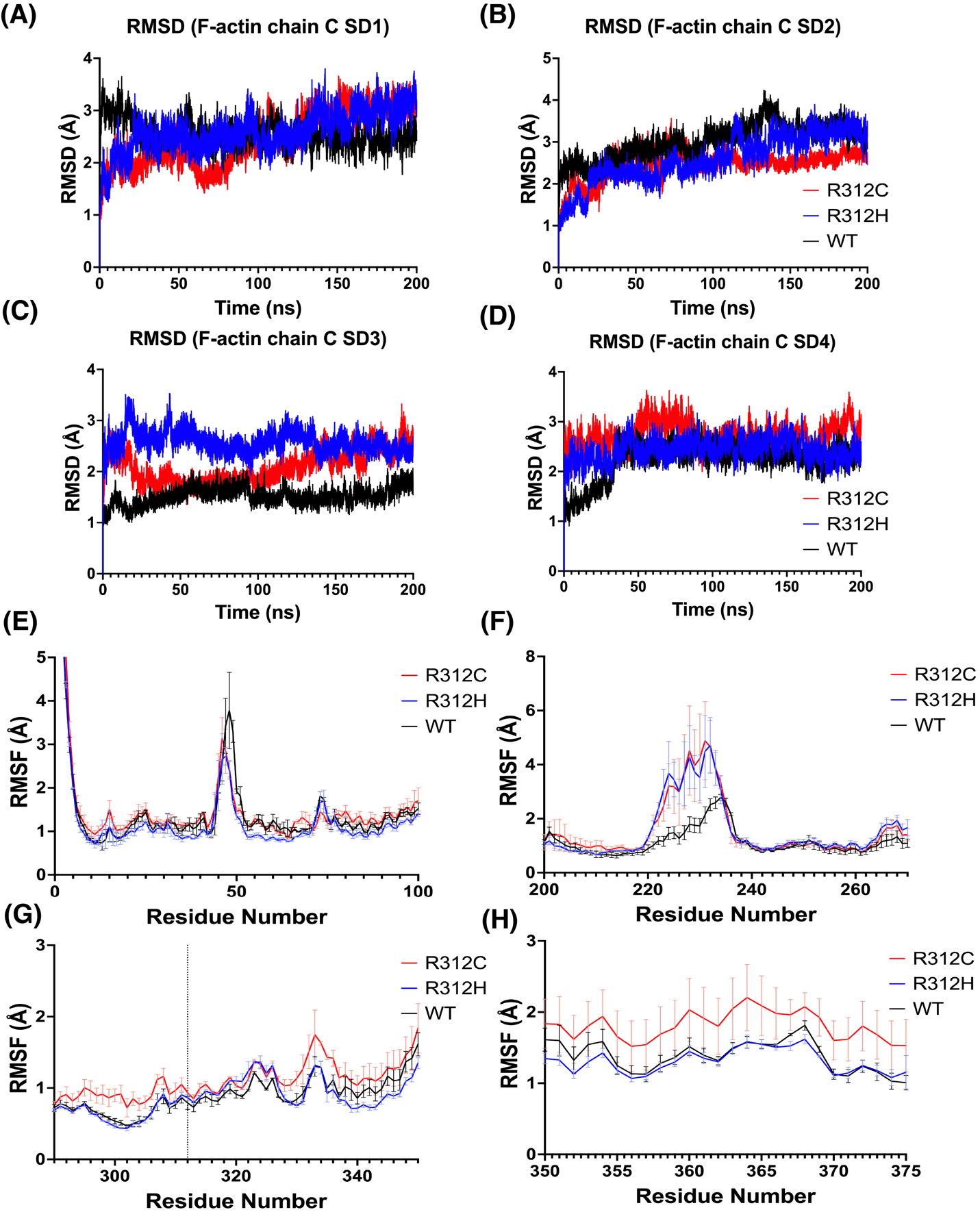
**

**Figure S2 Structural properties of F-actin protomers.** Additional details on changes to structural properties in F-actin simulations are shown. **(A)** Average RMSD plot (N=3) for actin SD1 shows a similar magnitude of changes between systems relative to the starting structure. **(B)** Average RMSD plot (N=3) for actin SD2 shows a similar magnitude of changes between WT and R312H, but fewer changes in R312C relative to the starting structure. **(C)** Average RMSD plot (N=3) for actin SD3 shows that both R312C and R312H variants exhibit greater differences in SD3 than WT when compared to the starting structure. **(D)** Average RMSD plot (N=3) for actin SD4 shows a similar magnitude of changes between systems when compared to the starting structure. **(E)** Average RMSF plots (N=3) for residues 1-100 for WT, R312C, and R312H, with error bars representing SEM. Both R312C/H variants show reduced flexibility around D-loop residues 45-50. **(F)** Average RMSF plots (N=3) for residues 200-270 for WT, R312C, and R312H, with error bars representing SEM. Both R312C/H variants show increased flexibility around the Tm-bumper (residues 222-230) as well as increased flexibility around the hydrophobic plug (residues 262-274). **(G)** Average RMSF plots (N=3) for residues 290-350 for WT, R312C, and R312H, with error bars representing SEM and a vertical dotted line at residue 312. R312C exhibited increased flexibility throughout the region. R312H displayed increased flexibility around tropomyosin binding residues 320-330, with decreased flexibility around residues 340-350. **(H)** Average RMSF plots (N=3) for residues 350-375 for WT, R312C, and R312H, with error bars representing SEM. R312C exhibited increased flexibility throughout this region.

**
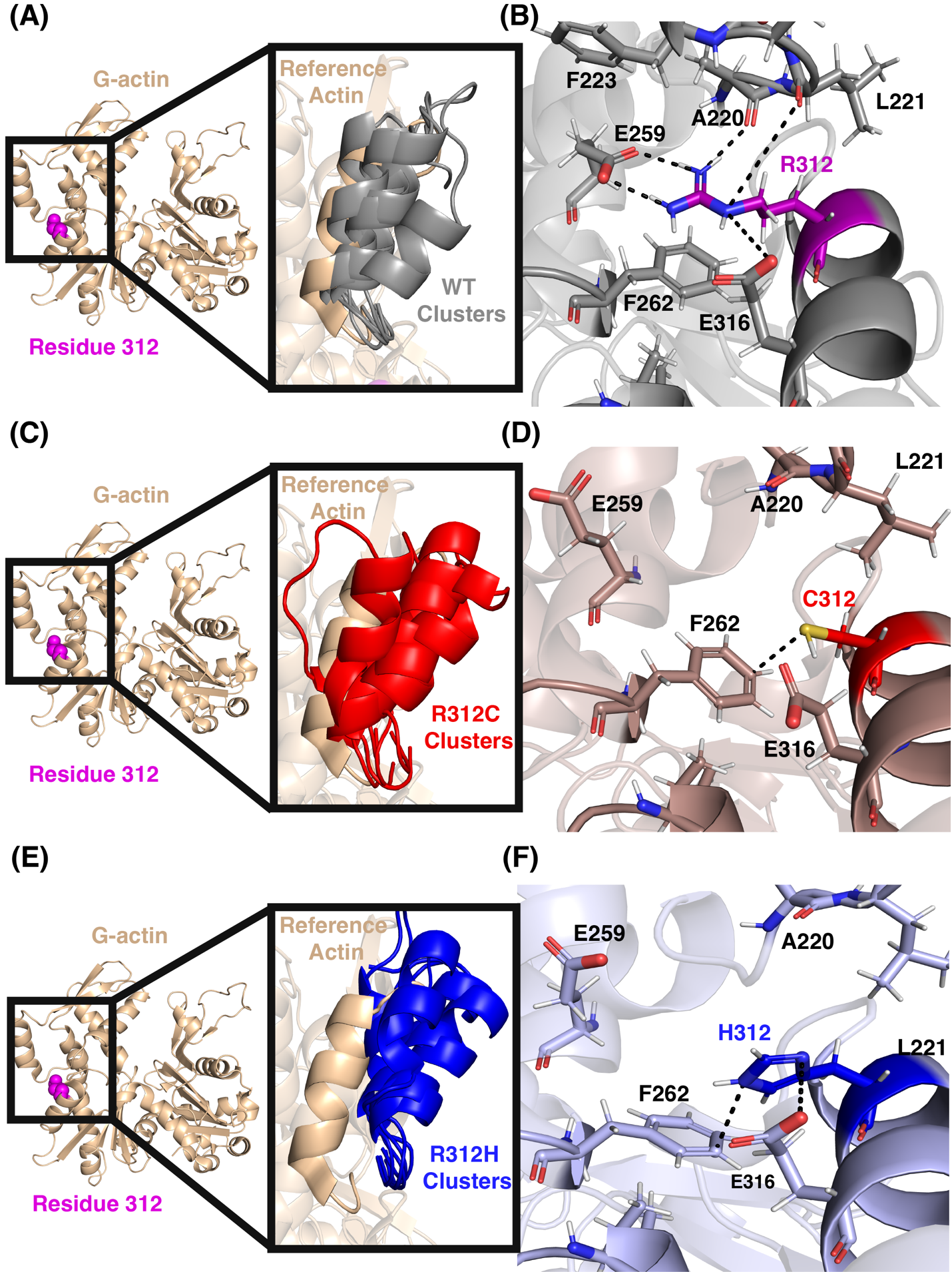
**

**Figure S3** **G-actin structural clusters.** The 5 largest structural clusters for each G-actin system were isolated, and a structural superposition was performed to the starting structure. **(A)** The monomeric structure (coloured wheat) with residue R312 (coloured pink) shown with its side chain as spheres. The sub-panel shows the range of motion for the WT structural clusters (coloured grey) around the Tm-bumper (residues 222-230). **(B)** In the largest WT structural cluster, residue R312 maintains stabilizing interactions (represented by dotted black lines) with nearby residues including the main chain of A220 at the bottom of the Tm-bumper. **(C)** The monomeric structure (coloured wheat) with residue C312 (coloured pink) shown with its side chain as spheres. The sub-panel shows the range of motion for the R312C structural clusters (coloured red) around the Tm-bumper (residues 222-230). Forward and backward shifts of the Tm-bumper are observed. **(D)** In the largest R312C structural cluster, stabilizing interactions between C312 and neighbouring residues are lost, instead associating with F262. The loss of stabilizing interactions with A220 allows the Tm-bumper to exhibit large shifts in its conformation. **(E)** The monomeric structure (coloured wheat) with residue H312 (coloured pink) shown with its side chain as spheres. The sub-panel shows the range of motion for the R312H structural clusters (coloured blue) around the Tm-bumper (residues 222-230). A forward tilt of the Tm-bumper is observed. **(F)** In the largest R312H structural cluster, stabilizing interactions with A220 are lost as H312 instead associates with F262 and E316, though weak interactions with A220 are possible depending on orientation.

**
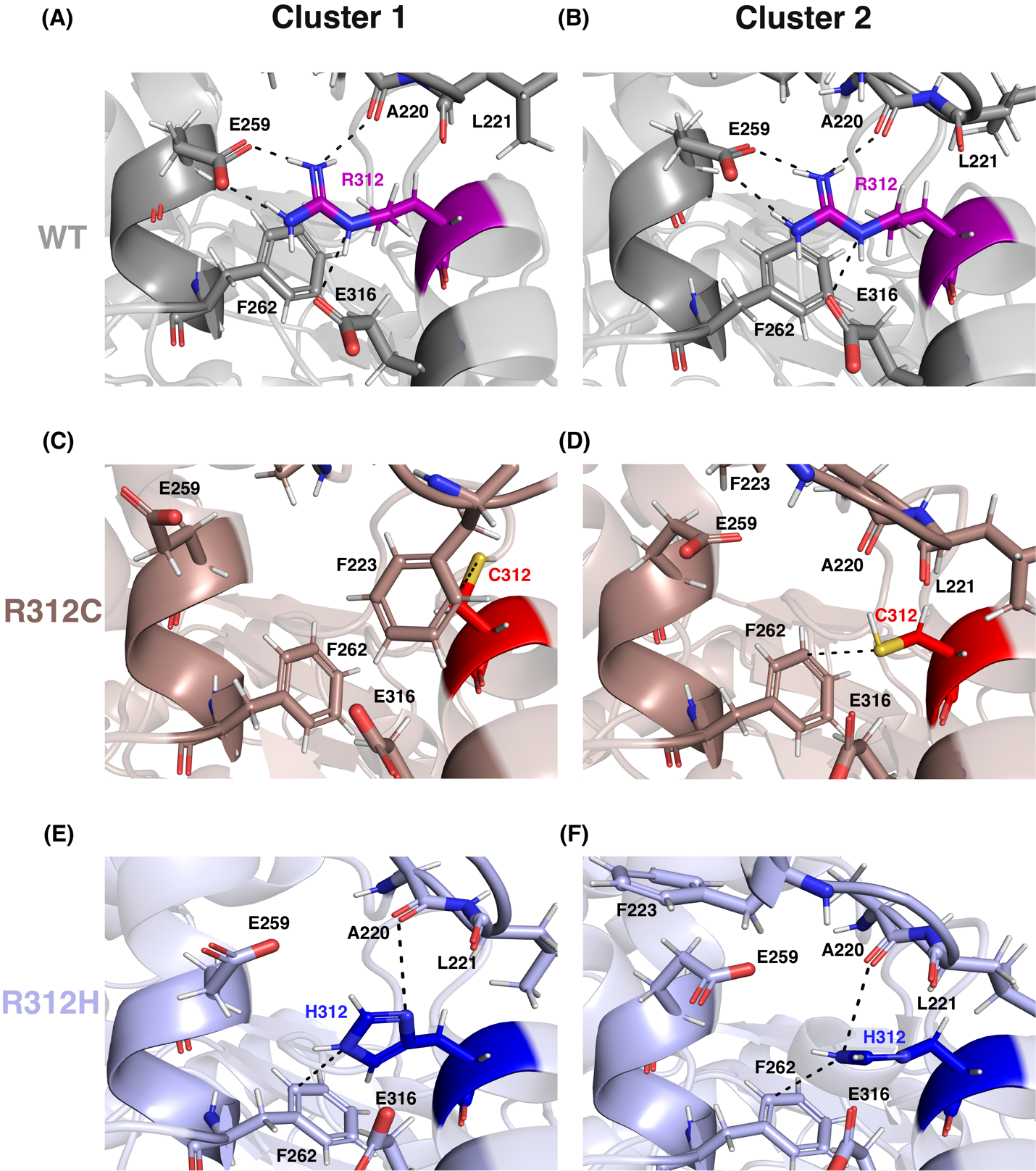
**

**Figure S4** **Residue 312 stabilizing interactions in F-actin structural clusters. (A)-(B)** In the two largest WT F-actin structural clusters (coloured grey), R312 (coloured magenta) maintains stabilizing interactions with residues A220, E259, and E316, restricting the Tm-bumper to a narrow range of motion. **(C)-(D)** In the two largest R312C F-actin structural clusters (coloured raspberry), C312 (coloured red) loses its stabilizing interactions with A220, E259, and E316. In the largest cluster, the Tm-bumper swings forward into a position stabilized by F223 coming into contact with C312. In the second largest cluster, C312 associated with F262, allowing the Tm-bumper to maintain its large range of motion. **(E)-(F)** In the two largest R312H F-actin structural clusters (coloured light blue), H312 (coloured dark blue) associates with F262. Dependent on its geometry, H312 can associate with E316 or form weak stabilizing interactions with A220. The reduced stabilizing interactions between H312 and A220 allows the Tm-bumper to shift forwards, similar to R312C. The possibility of H312:A220 stabilizing interactions, however, limits the frequency of Tm-bumper shifts when compared to R312C.

**
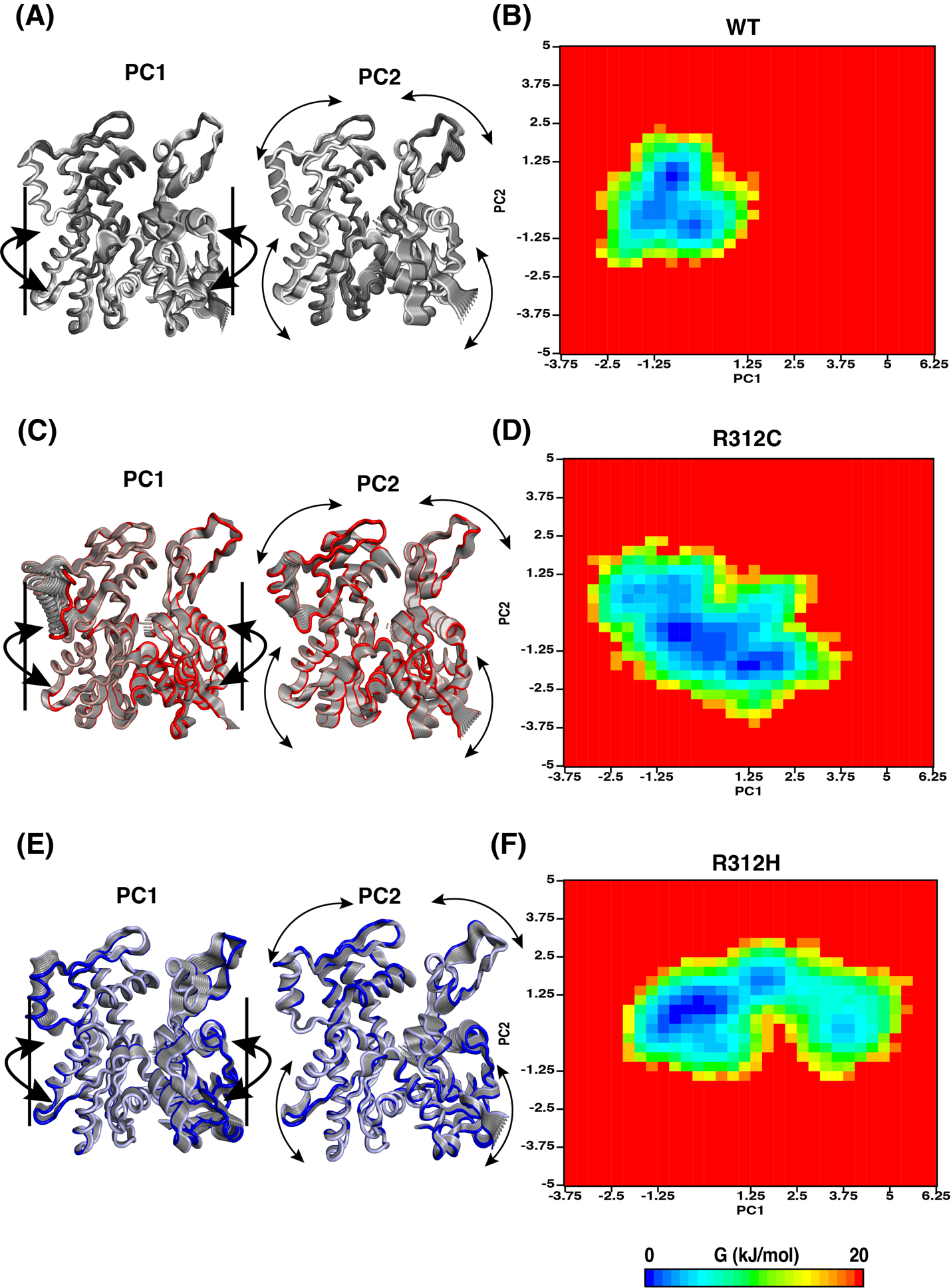
**

**Figure S5 F-actin protomer PCA.** Principal components analysis (PCA) was performed on F-actin chain C. **(A)** The extreme motions along the two largest principal components (PC1, PC2) were projected onto the WT protomer, with the largest range of motion occurring in SD1. **(B)** The WT Gibbs free energy landscape (FEL) was calculated along PC1/PC2 coordinates based on the number of simulation timepoints accessing those coordinates. Red represents limited access to those coordinates, indicating an energetically unfavourable conformation, while dark blue represents a large number of timepoints accessing those coordinates, indicative of an energetically favourable conformation. The WT protomer is defined by a single, small energy basin, indicative of a narrow range of conformations accessed. **(C)** Projection of the extreme PC1/PC2 motions onto the R312C protomer. Relative to WT, an increased range of motion is observed in the Tm-bumper. **(D)** The FEL along PC1/PC2 was calculated for the R312C protomer, indicating a larger energy basin relative to WT, indicative of a wider range of conformations sampled. **(D)** Projection of the extreme PC1/PC2 motions onto the R312H protomer. Relative to WT, an increased range of motion is observed in the Tm-bumper and SD2 around the D-loop. **(D)** The FEL along PC1/PC2 was calculated for the R312H protomer, indicating a larger energy basin relative to WT, indicative of a wider range of conformations sampled. The R312H energy basin spans the coordinates sampled by WT, with two smaller energy minima further along PC1.

**
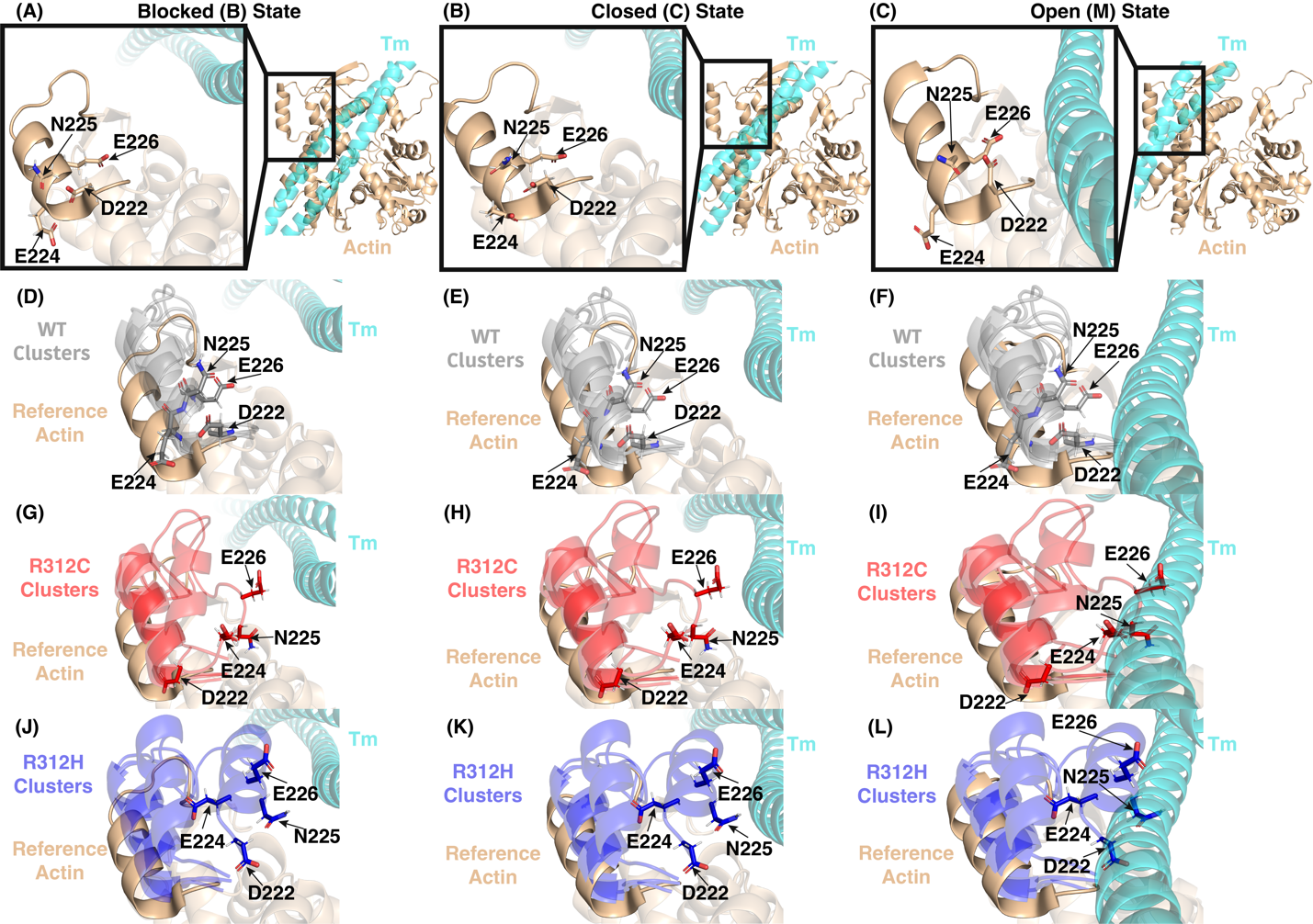
**

**Figure S6 The impact of actin structural clusters on tropomyosin binding. (A)-(C)** Tropomyosin (coloured cyan) overlayed on an actin protomer (coloured wheat) to show tropomyosin’s positioning in the blocked (PDB 7UTL), closed (PDB 7UTI), and open (PDB 8EFI) states. A sub-panel shows the position of the Tm-bumper relative to each tropomyosin conformation. **(D)-(F)** The five largest WT structural clusters (coloured grey) are aligned to a reference protomer (coloured wheat) via structural superposition. The Tm-bumper and it's position relative to tropomyosin in its blocked, closed, and open states is shown. The Tm-bumper is maintained in a restricted range of motion, with residues D222, N225, and E226 able to interact with Tm in its open state conformation. **(G)-(I)** The five largest R312C structural clusters (coloured red) are aligned to a reference protomer (coloured wheat) via structural superposition. The Tm-bumper and it's position relative to tropomyosin in its blocked, closed, and open states is shown. Forward shifts of the Tm-bumper bring E224, N225, and E226 into close proximity with tropomyosin in its blocked and closed conformations, forming stable interactions not present in WT. The Tm-bumper sterically interferes with the open state conformation, reducing activity under high calcium conditions. **(J)-(L)** The five largest R312H structural clusters (coloured blue) are aligned to a reference protomer (coloured wheat) via structural superposition. Similar motions of the Tm-bumper as R312C are observed, bringing it into contact with tropomyosin’s blocked and closed states while sterically interfering with the open state conformation. The result is reduced activity under high calcium concentrations *in vitro*, similar to what is observed with R312C.

**
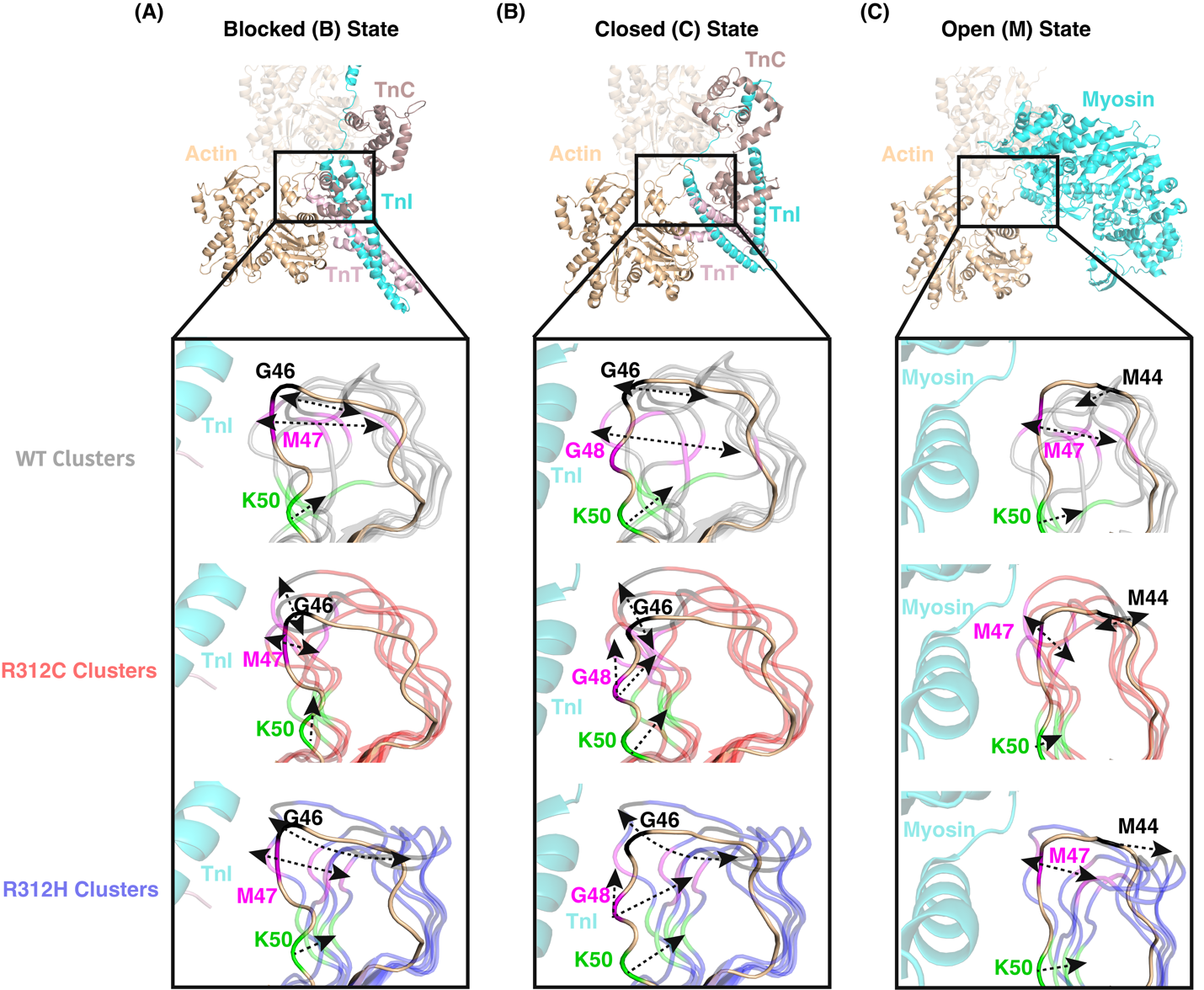
**

**Figure S7 The impact of actin structural clusters on myosin and troponin binding. (A)** Troponin in the blocked state conformation (PDB 7UTL) was isolated. A structural superposition overlayed the five largest structural clusters for WT (grey), R312C (red), and R312H (blue) with an actin protomer from PDB 7UTL (coloured wheat). **(B)** Troponin in the closed state conformation (PDB 7UTI) was isolated. A structural superposition overlayed the five largest structural clusters for WT (grey), R312C (red), and R312H (blue) with an actin protomer from PDB 7UTI (coloured wheat). Troponin I (coloured cyan) interacts directly with the D-loop in the blocked and closed states, shown here. **(C)** Myosin in the open state (PDB 8EFI) was isolated. A structural superposition overlayed the five largest structural clusters for WT (grey), R312C (red), and R312H (blue) with an actin protomer from PDB 8EFI (coloured wheat). Myosin (coloured cyan) directly interacts with the D-loop, shown here. The WT D-loop exhibits a range of conformations, reflected by its increased flexibility in simulations. The R312C D-loop, which appears more stable throughout the simulations, exhibits a consistent shift ‘forwards’ towards TnI in both states as well as myosin in the open state. The R312H D-loop exhibits a consistent shift ‘backwards’ away from TnI and myosin. The proximity of the D-loop to TnI or myosin likely influences the strength and stability of these interactions, affecting force generation and the regulation of contractile activity.

**
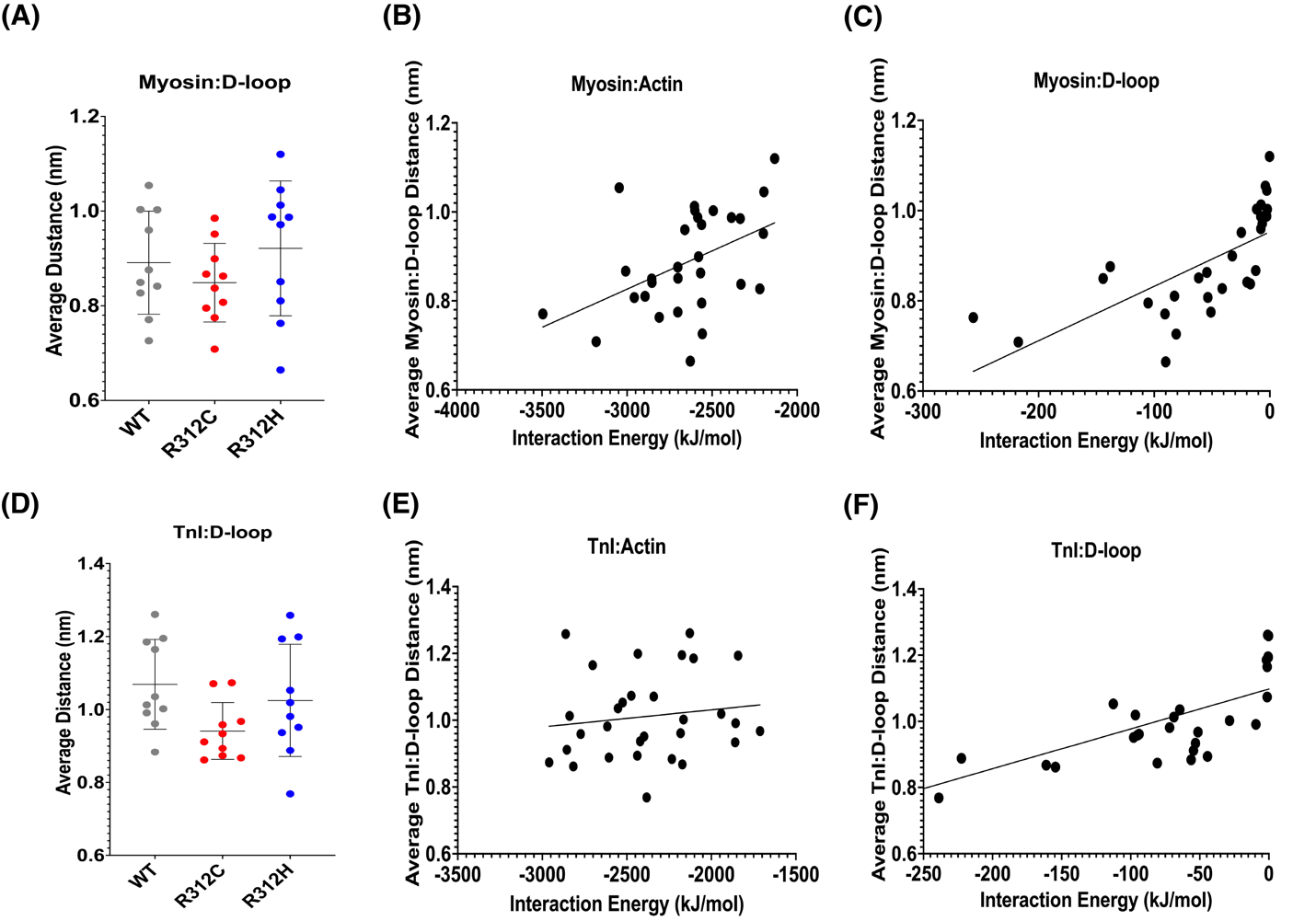
**

**Figure S8 D-loop positioning and its correlation with interaction energy.** D-loop positioning and its proximity to ABPs troponin and myosin appears to differentiate R312C and R312H variants. For all structures used for energy calculations, the minimum distance between each D-loop residue (residues 38-53) and TnI or myosin was calculated and averaged (N=10 where N is an actin structural cluster) to produce an average distance between the D-loop and either TnI or myosin. **(A)** The average minimum distance between myosin and all D-loop residues is plotted as a scatter plot for WT (grey), R312C (red), and R312H (blue), with a horizontal line at the mean and error bars showing SD. R312C exhibits a lower average distance than WT, while R312H exhibits a higher average distance than WT. **(B)-(C)** A correlation analysis was performed to determine if average D-loop proximity influenced the overall interaction energy. A scatter plot of the D-loop distance and corresponding interaction energy is plotted, with a line representing linear regression. Strong, statistically significant correlations were observed for D-loop distance and the myosin:actin interaction energy (r=0.4641 and p=0.0098) as well as the myosin:D-loop interaction energy (r=0.6848 and p<0.0001). **(D)** The average minimum distance between TnI and all D-loop residues is plotted as a scatter plot for WT (grey), R312C (red), and R312H (blue), with a horizontal line at the mean and error bars showing SD. R312C exhibits a lower average distance than WT, while R312H exhibits a higher average distance than WT. **(E)-(F)** A correlation analysis was performed. A scatter plot of the D-loop distance and corresponding interaction energy is plotted, with a line representing linear regression. A small correlation was observed between D-loop distance and the interaction energy between TnI and the entire actin filament (r=0.1359, p=0.4738). A strong, statistically significant correlation was observed for D-loop distance and the TnI:D-loop interaction energy (r=0.7000 and p<0.0001).
