# Supplementary material for: Duality in disease: How two amino acid substitutions at actin residue 312 result in opposing forms of cardiomyopathy
Source: J Biol Chem. 2024 Nov 5;300(12):107961. doi: 10.1016/j.jbc.2024.107961 (PMC11652881; doi:10.1016/j.jbc.2024.107961)
Supplement: Supplemental Tables S1–S7 [file mmc2.docx]

**Table S1 MD simulation setup.** A summary of all simulations conducted. G-actin models were generated based on the 2BTF reference structure. G-actin was simulated with ATP and Mg^2+^ in triplicate for 200 ns per replicate. F-actin models comprising 5 protomers were generated based on the 8A2S reference structure. F-actin was simulated with ADP-Pi and Mg^2+^ in triplicate for 200 ns per replicate.

|  |  | Reference Structure | Simulations  (replicates x simulation time) | Number of Protomers | Nucleotide State | Cation |
| --- | --- | --- | --- | --- | --- | --- |
| G-actin | WT | 2BTF | 3x200 ns | 1 | ATP | Mg^2+^ |
|  | R312C | 2BTF | 3x200 ns | 1 | ATP | Mg^2+^ |
|  | R312H | 2BTF | 3x200 ns | 1 | ATP | Mg^2+^ |
| F-actin | WT | 8A2S | 3x200 ns | 5 | ADP-Pi | Mg^2+^ |
|  | R312C | 8A2S | 3x200 ns | 5 | ADP-Pi | Mg^2+^ |
|  | R312H | 8A2S | 3x200 ns | 5 | ADP-Pi | Mg^2+^ |

**Table S2 Actin structures for interaction energies.** To calculate interaction energies, myosin, Tm, and Tn, were isolated from reference structures 8EFI, 7UTL, and 7UTI using the chains listed below. ABPs were individually docked to actin filaments comprising a variable number of actin protomers to ensure complete ABP binding.

| Protein | State | Reference Structure | Reference Structure Chains Used | Number of Actin Protomers |
| --- | --- | --- | --- | --- |
| Myosin | Open | 8EFI | M | 5 |
| Tm | Blocked | 7UTL | i+j | 11 |
|  | Closed | 7UTI | i+j | 11 |
| Tn | Blocked | 7UTL | U+V+Y | 8 |
|  | Closed | 7UTI | U+V+Y | 6 |

**Table S3 Actin structural clusters.** The size of each structural cluster, representing percentage of simulation time, is listed for G- and F-actin simulations of WT, R312C, and R312H systems.

|  |  | G-actin  (% of simulation time) | F-actin  (% of simulation time) |
| --- | --- | --- | --- |
| WT | Cluster1 | 9% | 16% |
|  | Cluster2 | 6% | 14% |
|  | Cluster3 | 6% | 9% |
|  | Cluster4 | 5% | 7% |
|  | Cluster5 | 4% | 6% |
|  | Cluster6 | 3% | 5% |
|  | Cluster7 | 2% | 4% |
|  | Cluster8 | 2% | 4% |
|  | Cluster9 | 2% | 3% |
|  | Cluster10 | 2% | 3% |
| R312C | Cluster1 | 9% | 6% |
|  | Cluster2 | 6% | 5% |
|  | Cluster3 | 5% | 5% |
|  | Cluster4 | 5% | 5% |
|  | Cluster5 | 4% | 5% |
|  | Cluster6 | 4% | 5% |
|  | Cluster7 | 4% | 5% |
|  | Cluster8 | 3% | 5% |
|  | Cluster9 | 3% | 4% |
|  | Cluster10 | 2% | 4% |
| R312H | Cluster1 | 11% | 6% |
|  | Cluster2 | 11% | 6% |
|  | Cluster3 | 8% | 5% |
|  | Cluster4 | 7% | 4% |
|  | Cluster5 | 4% | 4% |
|  | Cluster6 | 4% | 4% |
|  | Cluster7 | 3% | 4% |
|  | Cluster8 | 3% | 4% |
|  | Cluster9 | 3% | 3% |
|  | Cluster10 | 3% | 3% |

**Table S4** **Actomyosin Interaction Energies**. Interaction energies between myosin and an actin filament, as well as interacting actin DNaseI binding loops (D-loops, residues 38-53) and a section of subdomain 3 (residues 326-351) were calculated for the ten largest structural clusters across all three replicates (N=10). All energy values are reported in kJ/mol +/- SD. The average interaction energy between myosin and the actin filament was slightly more favourable for WT than R312C/H variants. The average interaction energy between myosin and the interacting D-loops was more favourable in R312C than in WT and R312H. While R312H had an average D-loop:myosin energy value similar to WT and R312C, the average interaction energy is heavily influenced by one cluster where the D-loop assumed a conformation similar to the R312C variant, reflected by the large standard deviation.

|  | WT Average (kJ/mol) | R312C Average (kJ/mol) | R312H Average (kJ/mol) |
| --- | --- | --- | --- |
| Filament - Myosin | -2748 ± 347 | -2642 ± 322 | -2550 ± 246 |
| D-loop - Myosin | -54 ± 56 | -57 ± 63 | -52 ± 80 |
| SD3 - Myosin | -667 ± 180 | -549 ± 133 | -530 ± 110 |

**Table S5** **Actin:Troponin Binding Energies**. Interaction energies were calculated for the ten largest structural clusters across all three replicates (N=10) between troponin’s Blocked/Closed states and an entire actin filament, troponin I (TnI) and an actin filament, as well as TnI and any directly interacting D-loops. All energy values are reported in kJ/mol +/- SD. In the Blocked state, Tn:F-actin and TnI:F-actin interaction energies were more favourable in R312C/H variants relative to WT. TnI:D-loop interaction energies were also substantially more favourable for both variants relative to WT, however, R312H interaction energy is largely influenced by the cluster demonstrating a forward shift of the D-loop similar to R312C. For the R312C/H variants in the Closed state, Tn:F-actin and TnI:F-actin interaction energies were less favourable relative to WT, while TnI:D-loop interaction energies were more favourable relative to WT.

|  |  | WT Average (kJ/mol) | R312C Average (kJ/mol) | R312H Average (kJ/mol) |
| --- | --- | --- | --- | --- |
| Blocked (B) State | Filament – Tn | -3266 ± 423 | -3566 ± 568 | -3398 ± 247 |
|  | Filament – TnI | -2294 ± 304 | -2440 ± 427 | -2403 ± 306 |
|  | D-loop - TnI | -33 ± 35 | -70 ± 55 | -112 ± 103 |
| Closed (C) State | Filament – Tn | -2620 ± 262 | -2508 ± 199 | -2495 ± 361 |
|  | Filament – TnI | -1244 ± 251 | -1229 ± 186 | -1201 ± 173 |
|  | D-loop - TnI | -135 ± 106 | -177 ± 74 | -203 ± 70 |

**Table S6** **Actin:Tropomyosin Interaction Energies**. Interaction energies were calculated for the five largest structural clusters across all three replicates (N=10) between tropomyosin’s Blocked/Closed states and an entire actin filament, the bumper helix (residues 222-230), as well as a section of subdomain 3 (SD3) comprising residues 321-335. All energy values are reported in kJ/mol +/- SD. In the Blocked state, overall Tm:actin interaction energies were less favourable in R312C/H. The Tm bumper helix formed favourable interactions with tropomyosin’s blocked state due to forward shifts of the helix. Interaction energies between the main Tm binding site in actin SD3 were substantially less favourable in both R312C/H variants relative to WT. Relative to the blocked state, overall actin:Tm energies in the Closed state are more favourable for R312C/H variants and less favourable for WT. In the Closed state, Tm:Tm-bumper interaction energies are substantially more favourable for R312C/H relative to WT, while Tm:actin SD3 energies are more favourable for R312C and less favourable for R312H relative to WT.

|  |  | WT Average (kJ/mol) | R312C Average (kJ/mol) | R312H Average (kJ/mol) |
| --- | --- | --- | --- | --- |
| Blocked (B) State | Filament - Tm | -4173 ± 598 | -4192 ± 477 | -3943 ± 810 |
|  | Bumper Helix - Tm | -37 ± 63 | -233 ± 333 | -134 ± 191 |
|  | SD3 - Tm | -2881 ± 485 | -2943 ± 571 | -2378 ± 828 |
| Closed (C) State | Filament - Tm | -3634 ± 661 | -4698 ± 899 | -4469 ± 1350 |
|  | Bumper Helix - Tm | -139 ± 126 | -718 ± 705 | -518 ± 308 |
|  | SD3 - Tm | -1660 ± 405 | -1782 ± 319 | -1458 ± 487 |

**Table S7 Average D-loop distance per cluster.** To identify correlations between binding energies and D-loop conformation, the average minimum distance was calculated between each structural cluster’s D-loop (residues 38-53) and myosin as well as troponin I. Average distances are reported in nm +/- SD. The average distances were lowest in R312C, whose D-loop exhibits a shift towards myosin and TnI. The average distances were highest in R312H, whose D-loop predominantly shifts away from myosin and TnI.

|  | WT Average (nm) | R312C Average (nm) | R312H Average (nm) |
| --- | --- | --- | --- |
| D-loop - Myosin | 0.89 ± 0.11 | 0.85 ± 0.08 | 0.92 ± 0.14 |
| D-loop - TnI | 1.07 ± 0.12 | 0.94 ± 0.08 | 1.02 ± 0.15 |
